# Three-dimensional cellularization in chytrid fungi uses distinct mechanisms from those driving one- and two-dimensional cytokinesis in animals and yeast

**DOI:** 10.1101/2025.01.30.635136

**Published:** 2025-09-24

**Authors:** Edgar M. Medina, Mary W. Elting, Lillian Fritz-Laylin

**Affiliations:** 1 Department of Biology, University of Massachusetts, Amherst, MA, 01003, United States; 2 Cluster for Quantitative and Computational Developmental Biology and the Department of Physics, North Carolina State University, Raleigh, NC, 27695, United States; 3 Howard Hughes Medical Institute and the Department of Biology, University of Massachusetts, Amherst, MA, 01003, United States

## Abstract

Chytrid fungi provide a model for studying three-dimensional cellularization, where nuclei that are dispersed throughout the cytoplasm are synchronously compartmentalized into daughter cells. This organization poses geometric challenges not faced by cells undergoing conventional cytokinesis or *Drosophila* monolayer cellularization, where nuclei are organized in one- or two-dimensional arrangements. We use the chytrid *Spizellomyces punctatus* to show that chytrid cellularization begins with nuclei and centrosomes migrating to the plasma membrane, where centrosome-associated vesicles mark sites of membrane invagination. The resulting tubular furrows extend, creating a cellularization foam of polyhedral compartments, each with a nucleus and cilium. Using inhibitors and laser ablation, we show that actomyosin networks drive cellularization, while microtubules are important for patterning cellularization and enable ciliogenesis but are not essential. Finally, we suggest that chytrids may have incorporated ancestral mechanisms associated with ciliogenesis to coordinate the association of internal nuclei with membrane furrows to solve the unique challenges associated with three-dimensional cellularization.

## INTRODUCTION

**Figure 1.**
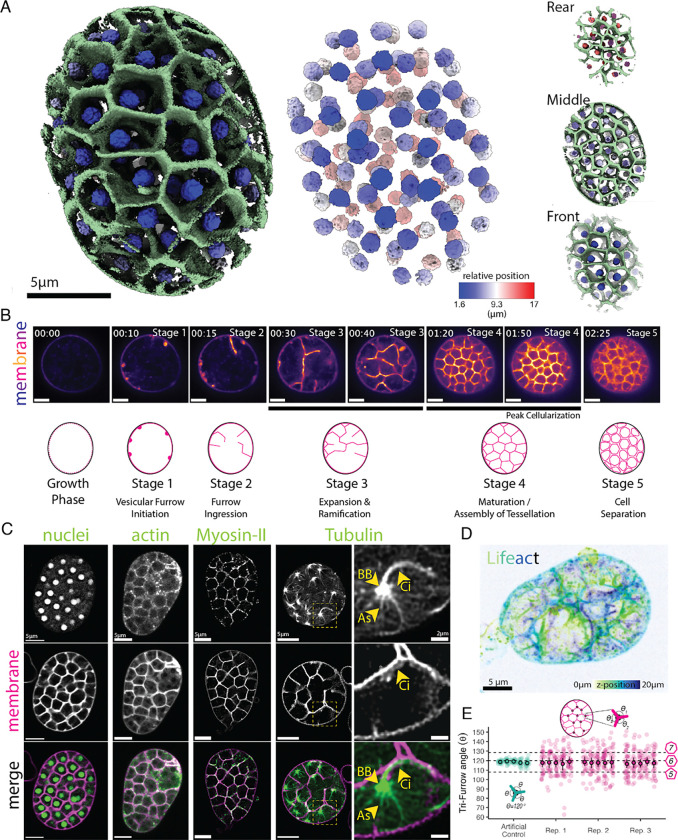
The chytrid *Spizellomyces* cellularizes by building a 3D tessellation of membrane polyhedra of homogeneous size, hexagonal symmetry, and demarcated by actin and myosin-II activity. **(A)** ChimeraX 3D reconstruction from a point scanner confocal live-cell image of a chytrid sporangium at peak cellularization (Stage 4). Membrane polyhedra are uninucleated and homogeneously distributed across the volume of the sporangium (front, middle, rear). FM4–64 dye was used for membrane (green) and nuclear-localized mClover3 for highlighting nuclei (blue to red). The nuclear position is shown as relative depth from the first stack image. **(B)** Images and idealized schematic of the five main stages of development of the membrane cleavage structures of chytrid cellularization. Note that during Stage 5—cell separation—cells remain packed and thus do not acquire a spherical shape until some cells escape the mother. **(C)** Actin and myosin-II colocalize with the membrane of the cellularization polyhedra. Live-cell confocal imaging of strains expressing the (nuclei) NLS-mClover3 to track nuclei (same image used in 3D reconstruction in A), the F-actin probe Lifeact-mClover3 (actin), the myosin-II probe MRLC-mClover3 (MRLC), and mClover3-α-Tubulin (Tubulin) to track microtubules. The magnified region of microtubules highlights the ciliary axoneme (Ci) that starts elongating after entry into cellularization, region containing the basal body (BB), and the astral microtubules and bundles (As). Membrane dyed with FM4–64. Scale 5 μm. **(D)** During mid cellularization, actin (and its associated membrane) form an heterogenous network of surfaces of complex 3D organization and geometry. Image shown is a serial Z-section at 0.39 μm intervals of a sporangium expressing the F-actin probe lifeact-mClover3 during early cellularization, color-coded by depth. **(E)**The cellularization polyhedra have internal vertices with angles with a mean that is in agreement with hexagonal symmetry (magenta) Since regular hexagons cannot form a polyhedron or tile a volume, faces with other symmetries such as pentagons and heptagons may be used to form the 3D tessellation and fill the volume of the mother cell. The angles were measured for at least twenty vertices within the central slice of a sporangium and for five sporangia in three biological replicates. These angles were compared to a set of five artificial vertices (teal) with perfect hexagonal symmetry (120 degrees) and similar cleavage membrane thickness and proportions to assess technical measurement error.

Animal and fungal cells divide by forming a trench-like membrane furrow that separates daughter nuclei into two distinct compartments (Glotzer, 2017; Pollard and O’Shaughnessy, 2019). This type of cytokinesis usually occurs immediately after nuclear division with the two daughter nuclei arranged in a straight line, which we therefore call a “one-dimensional” cytokinesis. However, when multiple rounds of nuclear division occur before cytokinesis, the resulting multinuclear cell state must be resolved by more complex cellularization processes. During *Drosophila* development, for example, hundreds of nuclei arrange themselves in a uniform monolayer near the plasma membrane, making the outcome of cellularization a two-dimensional sheet of cells at the surface of the embryo—“two-dimensional” cytokinesis (Sokac et al., 2023). Chytrid fungi represent an even more complex example that takes the geometry of cellularization to the third dimension (Fisher et al., 2000; Lowry et al., 2004; Lowry and Roberson, 1997). These fungi, which are key aquatic pathogens and important players in global carbon cycling, reproduce by compartmentalizing dozens of nuclei that are distributed homogeneously throughout the cytoplasm of a spherical mother cell surrounded by a cell wall—the sporangium (Freeman et al., 2009; Gleason et al., 2017; Kagami et al., 2014; Longcore et al., 1999; Martel et al., 2013; Medina and Buchler, 2020). Chytrid cellularization results in a ball—or morula—of uninucleated daughter cells of uniform volume, each bearing a motile cilium (Figure 1). The mechanisms that organize and drive this “three-dimensional” form of cellularization remain unclear.

Despite their different geometries, one-dimensional and two-dimensional cellularization involve the same four fundamental steps. First, nuclei find their positions. Second, the division site is selected. Third, the cell builds a cleavage structure at the division site. Fourth, the cleavage structure separates the mother cell into daughter cells. Animal and yeast cells use the same general strategies to complete these four steps. Both lineages rely on the pushing and pulling forces of microtubules to position their nuclei (Deshpande and Telley, 2021; Padilla et al., 2022; Xiang, 2018). Both use plasma membrane-anchored cues to define the position of the cleavage furrow (Glotzer, 2017; Pollard and O’Shaughnessy, 2019). Finally, both drive inward ingression of a trench-like furrow using a contractile ring made of actin and myosin-II (Glotzer, 2017; Pollard and O’Shaughnessy, 2019). These same strategies, however, cannot be extrapolated directly to three-dimensional cellularization for two reasons. First, interior nuclei are obstructed from access to cues placed on the plasma membrane. Second, it is not obvious how the contraction of actomyosin rings assembled at the plasma membrane could generate the many planes needed to surround the inner nuclei present during three-dimensional cellularization. Thus, it remains unclear whether and how the mechanisms used for conventional cytokinesis and two-dimensional cellularization apply to three-dimensional cellularization.

Multinuclear cellularization is common across many eukaryotic lineages (Hyde et al., 1991; Otegui and Staehelin, 2000; Shaw and Tilney, 1992), and its molecular mechanisms remain poorly understood (McCartney and Dudin, 2023). We here dissect the molecular mechanisms driving three-dimensional cellularization in the model chytrid fungus *Spizellomyces punctatus* (*Sp*) (Medina et al., 2020). We identify the main stages of cellularization and use pharmacological and physical perturbations to disrupt them. We find that the molecular mechanisms driving chytrid cellularization involve a combination of actomyosin machinery similar to that used for conventional cytokinesis along with ciliogenesis-like events not seen in either conventional cytokinesis or *Drosophila* cellularization. One-, two-, and three-dimensional cellularization therefore together represent a key example of different cellular events that use overlapping molecular machinery for the same objective via distinct molecular mechanisms.

## RESULTS

### Membranes colocalize with actin and myosin-II to form a three-dimensional cellularization lattice.

To begin, we confirmed that cellularization is indeed three-dimensional in *Spizellomyces punctatus* (*Sp*). To determine the spatial distribution of nuclei and membrane during peak cellularization—the stage in which the network of cellularization furrows occupies the full cytoplasm of the mother cell—we developed a method to grow synchronized cultures of *Sp* adhered to glass imaging dishes (see methods). With this system, we tracked nuclei and membranes using a native nuclear localization signal fused to the fluorescent protein mClover3 and the fluorescent membrane dye FM4–64. Using 3D point-scanner confocal imaging, we found that during peak cellularization, each nucleus is enveloped inside a polyhedron-shaped membrane (Figure 1A; Supplementary Video 1). These uninucleated polyhedra are tightly packed throughout the spherical sporangium—like soap bubbles inside a container—confirming that chytrid cellularization is indeed three-dimensional.

To determine how the cellularization lattice forms, we conducted time-lapse microscopy of FM4–64-stained membranes during cellularization and identified five main stages of membrane reorganization (Figure 1B). First, small, spherical invaginations form at the plasma membrane **(Stage 1)**. These “furrow initials” extend and elongate inwards toward the center of the cell, forming a membrane projection similar to a tubule that we call a “tubular furrow” **(Stage 2)**. (*Note*: *we recognize this term is an oxymoron but have chosen to retain the term “furrow” not because of its shape, but because of its historical context in the cytokinetic literature.)* These tubular furrows then invade the cytoplasm, branching and merging as they grow **(Stage 3)**, resulting in a tightly packed polyhedral network that occupies the entire sporangium **(Stage 4)**. Finally, in a single synchronous event of cell separation, called abscission, all of the polyhedra turn into individual daughter cells **(Stage 5)**. The resulting individual cells then swim and/or crawl out of one or more discharge pores in the sporangial cell wall, leaving behind the empty shell of their mother.

We next identified cellular machinery associated with the formation of the cellularization polyhedra. Animal and yeast cellularization relies on conserved cytoskeletal elements, primarily actin, myosin-II, and microtubules (Pollard and O’Shaughnessy, 2019). To explore the roles of these elements in chytrid cellularization, we visualized the distribution of actin networks with the actin polymer-binding probe Lifeact fused to the fluorescent protein mClover3 and co-stained cells with FM4–64. We found that during stage 4 peak cellularization, both actin and membrane localize to the polyhedral cellularization lattice (Figure 1C; Supplementary Video 2). We next determined the distribution of a fluorescently-tagged myosin regulatory light chain (MRLC)—a proxy for myosin-II activity (Heissler and Sellers, 2015). We found that, like actin, the fluorescent MRLC colocalizes with the membrane in the cellularization lattice, suggesting a possible role of contractile actomyosin in chytrid cellularization (Figure 1C; ; Supplementary Video 3). Finally, by expressing a fluorescently tagged α-tubulin, we observed three previously described microtubule structures closely associated with each nucleus during peak cellularization (Barron and Hill, 1974; Lessie and Lovett, 1968; Renaud and Swift, 1964). First, microtubule structures emanate from a focus in close apposition to each nucleus, structures that we infer to be the centriolar centrosome and basal body. Second, a long, bright and homogenous structure extending from the nuclear-associated basal body towards the nearest cleavage plane—the ciliary axoneme. Third, an heterogenous and variable network of astral microtubules that extend from the basal body and around the nucleus (Figure 1C Supplementary Video 4).

To explore how the tubular furrows of early cellularization transform into the polyhedral tessellation of late cellularization, we generated a volumetric image during furrow expansion and ramification using our actin probe (Stage 3) (Figure 1D). We found that the invaginating tubular furrows promptly acquire complex shapes that appear similar to minimal surfaces—smooth surfaces that minimize local surface area for a given boundary, like soap film in a wireframe—whose study lies at the interface of mathematics, geometry, and physics (Delsaulx, 1884; Plateau, 1873). Indeed, the surfaces and shapes we found in the developing sporangium are irregular and are more akin to melted cheese stretching between pizza and its pan than the orderly surfaces of the furrows formed during *Drosophila* cellularization (see discussion on foams and minimal surfaces). This volumetric imaging also highlights the three-dimensional and branched architecture of the cellularization furrows, explaining why actin or membrane structures appear bent in single imaging planes (e.g. Fig 1B).

To dissect in more detail the geometric patterning formed during chytrid cellularization, we measured the angles formed at the vertices of the cellularization polyhedra in sporangial cross sections (Figure 1E). A sampling of cross sections through a polyhedron gives a distribution of shapes that reflects the most common shapes of its faces. For example, cross sections though a triangular prism—which has five faces, three rectangular and two triangular—can create polygons with three, four, or five sides, with the majority of cross sections having four sides. Cross sections through the cellularization lattice displayed vertices with three furrows meeting at an angle with a mean of 117.8 degrees, consistent with zoospore territories comprising polygons with primarily pentagonal (108°) and hexagonal (120°) faces. This arrangement is likely a reflection of the fact that regular hexagons cannot, by themselves, create a polyhedron or tile a volume. These results suggest that 3D cellularization in chytrids entails the generation of a foam-like tessellation of polyhedral surfaces. Finally, to measure the consistency of cells formed by this tesselation, we measured the diameters of the daughter zoospores released after cellularization and found a coefficient of variation of 17% (Supplementary Figure 1). This variation is similar to the variance in cell size observed in age-matched fission yeast (6%) and budding yeast (17%) cells (Turner et al., 2012), indicating that chytrid cellularization must incorporate mechanisms for tight control of cell size.

Together, these results show that cellularization in chytrids may rely on similar cytoskeletal elements as those used by animal and yeast cells and that the assembly of the cleavage structures are initiated at the plasma membrane, much like in yeast or animal cells. However, unlike animals and yeast, chytrids do not use trench-like furrows. Instead, chytrid furrows form as individual vesicles in the plasma membrane that extend into tubules that invade, merge, and weave together to form a complex tessellation of membrane polyhedra of homogenous volume that are enriched in pentagonal and hexagonal faces.

### Nuclear migration to the plasma membrane precedes the onset of cellularization.

**Figure 2.**
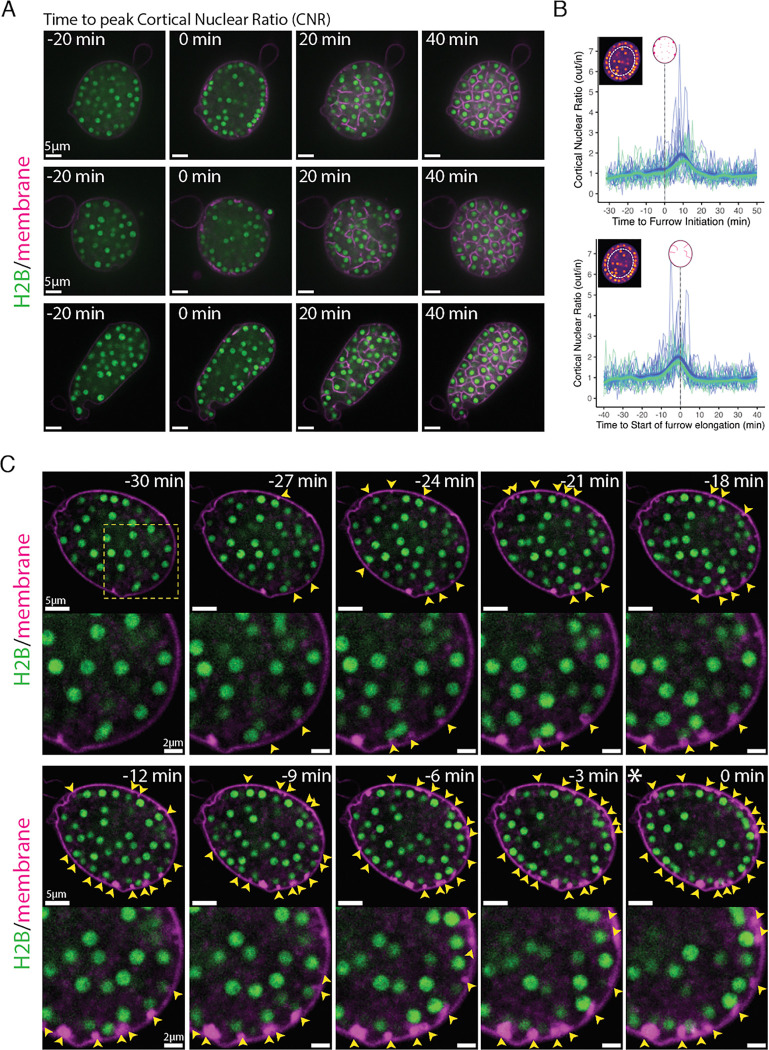
The onset of chytrid cellularization is marked by the recruitment of nuclei to the plasma membrane and the formation of vesicular membrane structures near cortical nuclei. **(A)** Chytrid nuclei (H2B-tdTomato) are homogeneously distributed across the sporangium before (−20 min) and after (+20 min) entry into cellularization except for a narrow window just at entry into cellularization (0 min; asterisk; coincides with the formation of the first membrane vesicles; Stage 1) when nuclei are enriched at the plasma membrane. Scale bars are 5 μm **(B)** Nuclear enrichment at the plasma membrane is limited to a narrow window of time (~10 min) at the onset of cellularization. After, the nuclei return to be homogeneously distributed. Quantification of nuclear dynamics during time-lapse microscopy 40 min before and after entry into cellularization. The cortical nuclear ratio corresponds to the ratio of the number of nuclei in the outer 50% area (a donut) relative to the inner 50% area (disk)—see inset image. The ratio distribution during time-lapse imaging for each sporangium was centered on zero based on the peak cortical nuclear ratio—marked with an asterisk. Blue and green signify two different biological replicates with thin lines showing individual sporangia and thick lines indicating loess regressions (99% confidence interval; span=0.2). **(C)** Recruitment of nuclei to the plasma membrane during entry into cellularization coincides with the formation of membrane vesicular structures (yellow arrows) in the proximity of each nucleus. Progression of nuclear recruitment and concomitant vesicular furrow initiation shown until peak cortical migration (asterisk; 0 min). Main scale bars 5 μm, inset 2 μm.

In animal and yeast cytokinesis, as well as during *Drosophila* 2D cellularization, daughter nuclei remain stationary while cytokinetic furrows extend between or around them. Because nuclei remain highly dynamic during chytrid cellularization, it is unclear if nuclear position plays a role in defining the placement, number, and assembly of furrows. To explore possible coordination between nuclear movement and furrow formation, we imaged synchronized cultures marked for membranes with FM4–64 and for nuclei with a fluorescently tagged histone H2B. Imaging cross-sections through the center of sporangia revealed a possible enrichment of nuclei at the cell surface just before cellularization (Figure 2A; Supplementary Video 5 & 6). To quantify this observation, we calculated the ratio of nuclei in two regions of equivalent area: an outer donut at the surface of the sporangium and the remaining inner disk—the cortical nuclear ratio. This ratio was near one before and during cellularization, except for a short window of ten minutes during which nuclei are enriched in the cortical region (Figure 2A–B). To test whether peak cortical nuclear ratio coincides with early stages of cellularization, we aligned the ratio profile of individual sporangia relative to the time of first vesicular furrow initial formation (Stage 1, Figure 2B, top) or relative to the start of furrow elongation (Stage 2, Figure 2B, bottom). We found that cortical nuclear enrichment occurs around 10 minutes after the start of Stage 1 (Figure 2B, top), and coincides with the start of furrow elongation (Figure 2B, bottom). Furthermore, when we align sporangia by the time of maximum nuclear enrichment, most sporangia (22 out of 24, 92%) progress into furrow elongation (Stage 2) within five minutes (Supplementary Figure S1). Thus, entry into cellularization is marked by the recruitment of nuclei to the plasma membrane.

**Figure 3.**
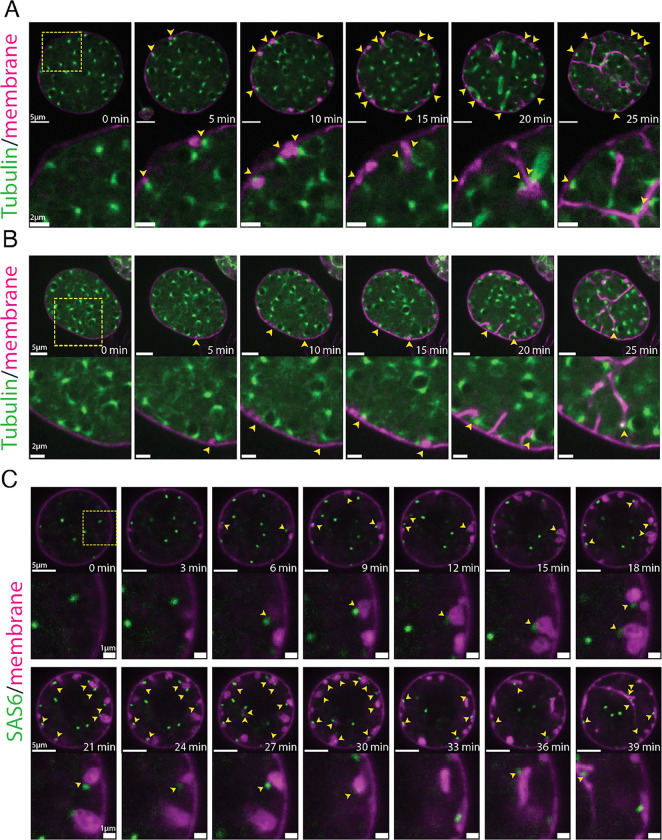
Cortical nuclei attach to the membrane vesicular furrow initials via the centrosomal region and are then dragged inwards when the vesicular furrows are elongated. **(A & B)** membrane vesicular furrows form in the proximity of the nuclear centrosomal regions (yellow arrows; 5-to-15min). The nuclei remain tethered to the membrane via the centrosomal region when the vesicular furrows extend inwards (15-to-25 min). Note that in (A) a nucleus forms a spindle and remains attached via the polar region of the spindle to the extending furrow. Main scale bars 5 μm, inset 2 μm. **(C)** A strain expressing a fluorescently tagged centrosomal component SAS6-mClover3 shows that the centrioles are proximal to the vesicular furrow initials (yellow arrows) and remain attached to the furrows during elongation. Membrane dyed with FM4–64. Main scale bars 5 μm, inset 1 μm.

To further explore the relationship between nuclear position and furrow initiation, we imaged at a higher frame rate and found that vesicular furrow initials form in proximity to the nuclei at the plasma membrane (Figure 2C). Imaging of the nuclei approaching entry into cellularization showed a progressive formation of vesicular furrow initials near cortical nuclei (Figure 2C). Because animal cells use astral microtubules to position furrows (Pollard and O’Shaughnessy, 2019), we hypothesized that patterning of chytrid cellularization may also involve microtubules. To test this hypothesis we followed cellularization in a strain expressing fluorescent tubulin (mClover3-alpha-Tubulin) in the presence of FM4–64 (Figure 3A & B; Supplementary Video 7–10). We found that each nucleus in an actively growing sporangium is associated with two opposing foci of tubulin fluorescence. Because these foci turn into spindle poles and duplicate immediately after nuclear division (Supplementary Figure S2), we infer that these foci represent centriolar centrosomes. Consistent with a role in furrow positioning for these microtubule organizing centers, we observed furrow initials forming in between the centrosomes and the plasma membrane (Figure 3A & B). To dissect in more detail the relationship between centrosomes and vesicular furrows, we built a strain expressing a fluorescently tagged version of SAS-6, a core component of eukaryotic centrioles (Ito et al., 2019). Cells expressing fluorescent SAS-6 show fluorescent foci at spindle poles and the base of the ciliary axoneme consistent with a centriolar distribution of SAS-6-mClover3. Upon entry into cellularization, SAS-6 puncta approach the plasma membrane, where they become associated with the furrow initials highlighted by FM4–64 (Figure 3C; Supplementary Video 11, 12, 24–26). The intimate association of centrioles with cleavage furrow initials agrees with ultrastructural evidence from the cellularization of the zoosporic fungus *Allomyces*, including electron micrographs that show the presence of basal bodies in close proximity to cleavage membranes as well as observations of nuclear movement coinciding with the start of cellularization (Fisher et al., 2000). These results suggest that arrival of the centrosomes to the plasma membrane precedes furrow initiation, a finding whose confirmation requires imaging at sub-minute resolution. Unfortunately, such experiments typically result in cells that fail to complete cellularization and die, likely due to phototoxicity (Supplementary Video 24). Nevertheless, our results show that during entry into cellularization, nuclei along with their associated centrosomes, are transiently recruited to the plasma membrane, where they associate with vesicular furrow initials.

To further understand the relationship between furrow formation and nuclear positioning, we followed the position of nuclei, centrosomes, and membrane through the later stages of cellularization. Consistent with centrosomes playing a major role in organizing chytrid cellularization, we found that during furrow elongation (Stage 2) the centrosomes appear to remain tethered to what seems to be the leading front of the furrow initials, dragging their nuclei with them (Figure 3C; Supplementary Video 11, 12, 25, 26). Taken together, these results suggest a physical connection between nuclei, centriolar basal bodies, and vesicular furrows throughout the cellularization process.

### Microtubules are important for geometric patterning but are not necessary for cellularization.

**Figure 4.**
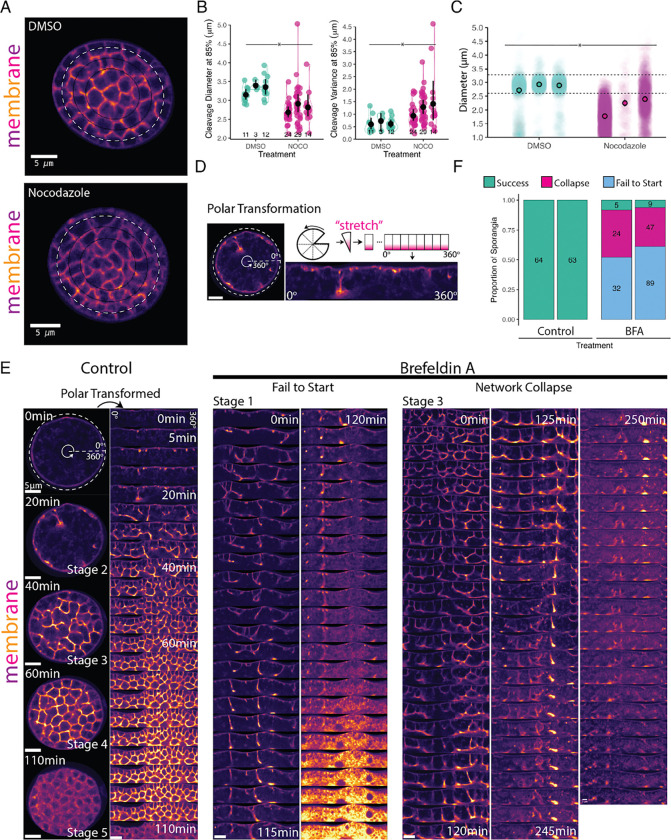
Depolymerization of microtubules does not inhibit cellularization but causes patterning defects and the membrane of cellularization is sourced primarily from the Golgi. **(A)** Sporangia treated with Nocodazole (2 μM) during cellularization causes a defect in the geometric patterning of cellularization but does not inhibit it. Concentric ellipses of 85% (white dashed line) and 65%, and 45% radii are shown in thin black lines **(B)** Daughter cells of sporangia treated with Nocodazole are smaller (left) and are more variable (right) in diameter than sporangia treated with the carrier control DMSO. The daughter size (cleavage diameter) was estimated using the distance between max-intensity peaks in a line scan of the fluorescence intensity of the membrane along the 85% radii ellipse. Cleavage planes at 85% tend to be perpendicular to the ellipse and thus more accurate. Significance was assessed by a permutation test of the difference of means. * p-value ≤ 0.05. **(C)** The effect of Nocodazole on zoospore size was confirmed by treating synchronous cultures of *Spizellomyces* with 2 μM Nocodazole for two hours upon entry to cellularization and measuring the size of the zoospores produced using a Coulter Counter. We found that Nocodazole causes a significant reduction in average zoospore size (permutation test of difference of means: * p-value ≤ 0.05). Although the variance appears to be affected, we found this difference not statistically significant (permutation test of difference of means, p-value=0.1). **(D)** Schematic of the polar transformation on an image of membrane organization (FM4–64) in a sporangium during cellularization. This transformation converts “circular” images with polar coordinates into “unwrapped” images in cartesian coordinates for easier visualization. In our case, the plasma membrane will be at the top and the furrows invade inwards towards the bottom. **(E)** Sporangia treated during cellularization with the inhibitor of ER-to-Golgi trafficking Brefeldin-A fail to complete cellularization because they either fail to start—furrows try to invade but fail to progress (left)—or cause collapse of the furrows already formed at the moment of treatment (right). In contrast, control sporangia treated with the carrier DMSO show a gradual increase in the complexity of patterning until completing cell separation (110 min). Bright FM4–64 fluorescence in the “fail to start” panel corresponds to cell death. **(F)** Quantification of the proportion of cells succeeding, failing to start, or collapsing during treatment with BFA or DMSO control. All scale bars are 5 μm.

Forces transmitted through microtubules help move and position nuclei and spindles during cytokinesis in animal and yeast systems (Burgess and Chang, 2005; Daga and Chang, 2005; Harvey, 1935; Rappaport, 1996, 1986). During *Drosophila* 2D cellularization, for example, microtubules move and position the nuclei in a monolayer under the plasma membrane in preparation for cellularization (Baker et al., 1993; Deshpande et al., 2022; Sokac et al., 2023). Furthermore, plus end-directed motors that ride on microtubules (Kinesin-6; Pav-KLP) may provide the driving force required during the early stages of furrow ingression and deliver via vesicles the actin and membrane required during *Drosophila* 2D cellularization (Minestrini et al., 2003; Sokac et al., 2023; Sommi et al., 2010). We therefore wondered if the microtubules nucleated from chytrid centrosomes play a similar role in furrow elongation. To test this hypothesis, we treated cellularizing sporangia of a chytrid strain expressing fluorescent tubulin with 2 μM Nocodazole. Unlike cells treated with the DMSO carrier alone, sporangia treated with Nocodazole showed rapid dissipation of all microtubule structures except for a bright dot at the centrosome (Supplementary Figure S3, Supplementary Video 23). Cells lacking microtubules formed cellularization polyhedra of uneven size with curved edges and rounded vertices (Figure 4A). Despite these patterning defects, Nocodazole-treated sporangia completed cellularization and produced daughter cells that lacked cilia (Supplementary Figure S3; Supplementary Video 23). In contrast, sporangia treated with DMSO alone assembled well-organized cellularization lattices (Figure 4A) and produced normal, ciliated daughter cells.

To quantify the patterning defect induced by microtubule depolymerization, we compared the size and variance of the cellularization polyhedra between Nocodazole-treated and control cells. To aid automated image analysis, we focused on the polyhedra contiguous to the sporangial cell wall because they have regular, perpendicular furrows (Figure 4A). We found that, while sporangia treated with DMSO produced polyhedra with diameters between 3–3.5 μm, sporangia treated with Nocodazole produced polyhedra between 2.5–3 μm—a 14% reduction in diameter, with an even greater change in variance (Figure 4B). We confirmed this finding by measuring the diameters of fully developed daughter zoospores by treating synchronous cultures with 2 μM Nocodazole upon entry to cellularization and harvesting the zoospores released after two hours of treatment (Figure 4C). We found a consistent and significant reduction in daughter size relative to DMSO control cells (diff of means one-tail permutation test=0.6973, p-value 0.05). Microtubules, therefore, are not strictly necessary for cellularization but do play an important role in patterning the membrane tessellation and regulating polyhedral/daughter size. Taken together with our previous data, this means that while the microtubule cytoskeleton is involved with attachment of nuclei to furrows (i.e. centrosomes), it does not play an active role in furrow invasion.

### The additional membrane needed for cellularization is sourced from the Golgi.

Building the three-dimensional cellularization lattice requires large amounts of membrane. Since the membrane used for cytokinesis in animals and yeasts comes from the Golgi, we wondered if this was also the case for chytrid cellularization. To test this hypothesis, we disrupted ER-to-Golgi trafficking with the drug Brefeldin A (BFA). We treated synchronous populations of *Sp* with either 50 μM BFA—or DMSO carrier control—in the presence of FM4–64 and followed sporangial development by time-lapse imaging for six hours (Figure 4D–F). To facilitate visualization of the cellularization pattern, we computationally “unwrapped” the circular sporangia by polar transformation (Figure 4D). While control sporangia completed cellularization within the imaging time window (N=127), only 7% of sporangia treated with BFA completed cellularization during the same period of time (N=206), 12 out of 14 of which had already started cellularization when BFA was added (Figure 4E). The sporangia that failed to cellularize displayed two main phenotypes. The first was “failure to start”. Sporangia with this phenotype were in either Stage 1 (pre-cellularization) or Stage 2 (with some level of furrow branching) upon BFA treatment (Figure 4E; Supplementary Video 13). Although the furrows of these cells made repeated movements toward the cell interior, these sporangia never progressed further in cellularization and eventually arrested cytoplasmic movement, vesiculated, and acquired intense, uniform FM4–64 fluorescence—suggestive of cell death. The second phenotype was “network collapse”, which occurred in sporangia that already had an advanced cellularization network (Stage 3) prior to BFA treatment (Figure 4E; Supplementary Video 14). The cellularization structures in these sporangia collapsed into a single disorganized bundle that gradually disappeared followed by a death similar to that seen in sporangia with the “failure to start” phenotype. Together, these results show that ER-to-Golgi trafficking is necessary for the formation and maintenance of *Sp* cellularization networks, in agreement with ultrastructural imaging of other chytrids, where cellularization coincides with hypertrophy of Golgi cysternae and vesicles as well as the proximity of these structures to cleavage furrows (Taylor and Fuller, 1981). Although we cannot reject additional contributions of other endomembrane systems to cellularization, the robustness and speed of our phenotypes suggests that the membrane for cellularization is sourced primarily from the Golgi.

### Actomyosin contraction is a driving force of 3D cellularization in chytrid fungi.

**Figure 5.**
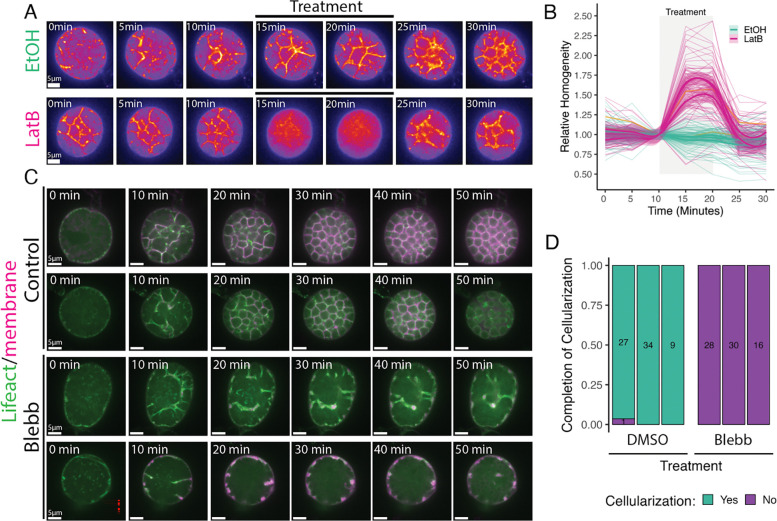
Chytrid cellularization requires actin polymerization and myosin-II activity. **(A)** Inhibition of actin polymerization with Latrunculin B (LatB) in a strain expressing the actin probe Lifeact-mClover3 causes disassembly of the actin cellularization network. This network is reassembled within 5 min after the washout of LatB. **(B)** Treatment with LatB (magenta) causes a sharp increase in sporangial homogeneity—reduction in texture, and thus disassembly of the actin network—that comes back to pre-treatment levels after the washout of LatB. Control sporangia (teal) treated with DMSO control shows no effect. The mean line and bootstrapping 95% confidence interval of the mean are shown for three biological replicates. Orange lines show the series shown in panel (A). **(C)** Treatment of sporangia with the inhibitor of myosin-II activity para-amino-blebbistatin (Blebb) inhibits cellularization. Control cells treated with DMSO control show normal cellularization, while sporangia treated with Blebb fail to advance beyond Stage 1 or 2. **(D)** Treatment of sporangia with Blebb completely inhibits cellularization. The box for each of the three biological replicates shows the number of sporangia analyzed per replicate. All scale bars are 5 μm.

Having shown that microtubules are not necessary for chytrid cellularization, we next asked whether cellularization requires dynamic actin structures. We began by treating cellularizing sporangia expressing a fluorescently-tagged F-actin probe (Lifeact) with Latrunculin B (LatB)—a reversible inhibitor of actin polymerization that sequesters actin monomers preventing their incorporation into actin filaments and accelerates depolymerization (Coué et al., 1987; Fujiwara et al., 2018). Treatment with LatB caused a complete disassembly of the actin cellularization networks within five minutes (Figure 5 A). This effect was reversible, as the cellularization networks began reforming within five minutes of LatB wash out, indicating that these networks are undergoing dynamic turnover. We quantified these phenotypes using fluorescence intensity variability (*i.e.* Haralick image texture) as a proxy for the structural complexity of the actin networks (Figure 5B). While sporangia treated with ethanol alone showed no significant change in texture, sporangia treated with LatB showed a 50–75% increase in homogeneity within five minutes of treatment. Washout of LatB returned texture levels to pre-LatB levels within five minutes. Because Latrunculin B inhibits new F-actin assembly, these results indicate that cellularization networks require active actin polymerization.

In animals and yeast, similar-looking actin networks collaborate with myosin-II motors to generate the contractile forces that drive cytokinesis. To test if myosin-II is also necessary for chytrid cellularization, we treated cellularizing sporangia expressing Lifeact with the myosin-II inhibitor para-amino-blebbistatin (blebbistatin). While sporangia treated with the carrier control DMSO underwent normal cellularization, sporangia treated with blebbistatin failed to complete cellularization (Figure 5C & D). These results show that both actin polymerization and myosin-II activity are required for chytrid cellularization.

The actin networks that drive cellularization in animals and yeast rely on myosin-II activity to generate tension. The contractile properties of these networks can be tested using pulsed lasers to ablate actomyosin structures in a diffraction-limited spot and observe their movements (Kassianidou et al., 2017; Kumar et al., 2006; Moshtohry et al., 2022; Silva et al., 2016; Spira et al., 2017; Wollrab et al., 2016). If chytrid cellularization is powered by a similar contractile mechanism, then severing the actin furrows should cause remaining structures to recoil as they release tension (Moshtohry et al., 2022; Silva et al., 2016; Spira et al., 2017; Wollrab et al., 2016). We therefore used laser ablation to test if the actin networks of both early cellularization furrows and late cellularization polyhedra are under tension.

**Figure 6.**
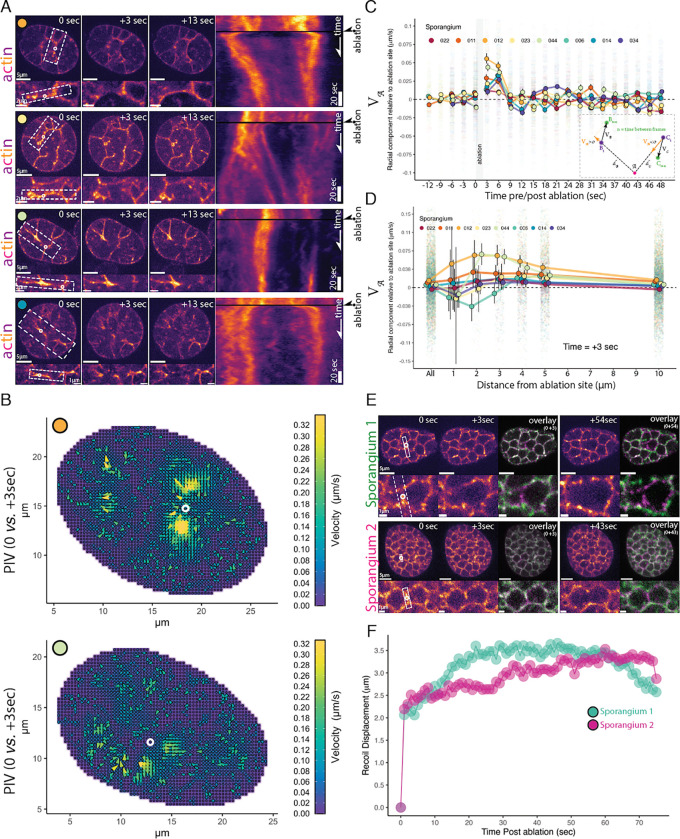
The actomyosin furrows of early cellularization and the edges of the late cellularization polyhedra are under tension. **(A)** Laser ablation of the actin furrows during early cellularization results in recoil. Four examples of sporangia ablation are shown. Colored circles correspond with sporangia shown in later panels. Each row shows still images of the sporangia just before laser ablation (0 sec, at white circle) and then 3 and 13 seconds after laser ablation, with dashed region inset below. The rectangle in each inset shows the 20-pixel wide selection used for the sum of intensity kymograph on the right. In each kymograph, the fast recoil response after the dark rows (ablation) is followed by contraction of the actin structure as if “repairing” the damaged region. Main scale bars 5 μm, inset scale bars 2 μm, 2 μm, and 1 μm, from top to bottom. **(B)** We used particle image velocimetry (PIV) to quantify the changes in the speed and direction of all actin structures of cellularization. Two examples (sporangia with corresponding color circles in panel A) of PIV velocity field plots comparing the frames before laser ablation (0 sec) and after ablation (3 sec). Note vectors with high velocity (warm color) moving away—recoiling—from the ablation sites (white circles). **(C)** Laser ablation causes a high velocity recoil away from the ablation site in all actin structures within 4 μm of the ablation site. We use the radial component of the velocity $\left({\text{V}}_{𝒜}\right)$ relative to the ablation site (𝒜) to show movement only in the direction of the ablation site (away—postive—or towards—negative; see cartoon in inset). The short period of negative ${\text{V}}_{𝒜}$ that follows the recoil in most sporangia is consistent with the “repairing” contraction towards the ablation site. Color dots show mean values (corresponding sporangia shown with the same color circles of A) and error bars show 99% confidence intervals of the mean by bootstrap. For visualization purposes we display data points within a range of −0.1 to 0.1 μm/s (this is 176417 points; 95% of the total data). **(D)** Radial component of the actin velocities $\left({\text{V}}_{𝒜}\right)$ relative to the ablation site (A) for vectors at different distances from the ablation site (*d* in cartoon) at three seconds after the start of ablation. For visualization purposes we display data points within a range of −0.15 to 0.15 μm/s (this is 23198 points; 97% of the total data). Note that highest velocities occur between 2–4 μm from the ablation site in most sporangia (see supplementary figures 4–6). **(E)** Laser ablation of the cleavage furrows during late cellularization also results in recoil and dilation of the polyhedral geometry. Overlay for frames before and the first frame after the end of ablation (3 sec from the “before” image) and between the frame before and the frame showing near maximal recoil displacement (54 sec & 43 sec). Main scale bars 5 μm, inset 1 μm. **(F)** Recoil displacement caused by ablation of the sporangia (pink and green) shown in E, along the direction indicated by the white dotted rectangle in the inset. The recoil of the polyhedral edges shows a sharp fast recoil of about ~2 μm—primarily from recoil of the cleavage furrow—followed by dilation of the surrounding polyhedra.

We first ablated furrows assembled during early cellularization (Stage 3), which caused large recoils of the actin structures (Figure 6A; Supplementary Video 15–20). Recoil after ablation was also observed in very early furrows (Supplementary Video 27). Following the initial recoil, we observed a tendency of the ablated structures to contract back into the ablated area (Figure 6A kymographs). This “repair” response is consistent with the response to ablation in other contractile actin networks (Moshtohry et al., 2022; Silva et al., 2016; Spira et al., 2017; Wollrab et al., 2016). To quantify the speed and direction of the recoil, we turned to particle image velocimetry (PIV) (Figure 6B–D). By using the pixels from the actin network images as tracer particles, we could account for the variability in response across cellularization structures in different sporangia. This analysis shows that, after laser ablation, many of the F-actin structures within a few μm of the damaged region move with high velocities away from the ablation site (Figure 6B), indicating that neighboring contractile structures likely distribute their load onto each other.

To characterize the time scale and spatial extent of the response to ablation, we further analyzed the radial component of the PIV motion, either away from (recoil) or toward (repair) the site of ablation at different distances from the targeted region (**see** Materials and Methods; Figure 6C, **inset**). Consistent with the qualitative observations above, this analysis demonstrates an initial recoil response—indicated by a positive radial component of the velocity lasting around nine seconds—followed by a short period of movement towards the ablation site consistent with possible repair of the damage structures—indicated by a negative radial component of the velocity (Figure 6C, Supplementary Figure 4). While we see some variation in timing and magnitude among different sporangia, this overall signal is robust to different strategies of pre-processing and sampling of the data (**see** Materials and Methods; Supplementary Figure 4–6). We found the fastest recoil signal occurred between two and four μm from the ablation site(Figure 6C, **Supplementary Figure 5**) and between three and six seconds after ablation (Figure 6D, **Supplementary Figure 6**). Due to the comparable timescales of recoil, ablation itself (~1–2 seconds), and our imaging frequency (1 Hz), there is significant uncertainty in the instantaneous magnitude of the recoil velocity, which may be faster than the ~75 nm/s we can detect (Figure 6C and D). However, the consistent recoil we observe for several frames following ablation shows that the actomyosin structures of early cellularization are indeed contractile, are under tension, and distribute their load over a distance of several μm.

Finally, we tested whether the F-actin polyhedra of late cellularization are also under tension. We observe a response that is similar in character as in early cellularization, but with faster recoil, suggesting higher tension by this stage. We found that laser ablation of the edges of the cellularization polyhedra resulted in a rapid recoil of the furrow tips away from the ablation site (Figure 6E; Supplementary Video 21 & 22), showing a displacement of approximately two μm within the first second after ablation followed by a shallow exponential recoil profile in which tension appear to release for more than 30 seconds (Figure 6F). After the first recoil, the two vertices at each side of the ablated furrow also recoiled away from each other, causing a dilation in adjacent polyhedra. Together, these results show that contractile actomyosin structures are used during furrow elongation as well as throughout the assembly of the cellularization polyhedra. Furthermore, F-actin network tension is important for establishing and maintaining the geometry of the cellularization network.

## DISCUSSION

**Figure 7.**
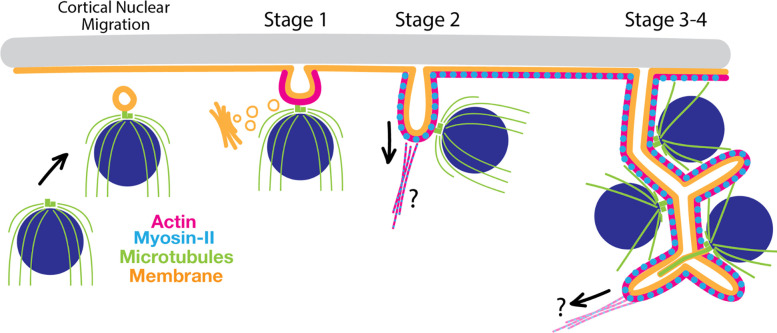
The model of chytrid 3D cellularization.(1) Nuclear cortical migration: Entry into cellularization is marked by the recruitment of nuclei and associated centrioles to the plasma membrane. We hypothesize that a “ciliary vesicle”-like structure may be associated with one centriole during nuclear migration. After migration, the centriolar centrosome of each nucleus faces the plasma membrane. **Stage 1, Vesicular furrow initiation:** the ciliary vesicle fuses to the plasma membrane—allowing the rapid diffusion of FM4–64 dye from the plasma membrane. This ciliary vesicle is also the vesicular furrow initial. This stage does not depend on myosin-II and the membrane sourced from this stage onwards is derived from the Golgi apparatus. **Stage 2, Furrow ingression:** Actin and myosin-II work together to drive the extension of the vesicular furrows into tubular furrows that invade the cytoplasm. Because the nuclei remain attached to their furrows via the centrosomal region, the extension inwards of the furrows drags the nuclei with them into the cytoplasm. We infer that nuclei dock during furrow initiation (Stage 1) because they move with the furrows during furrow ingression. The location and organization of the connections in the actomyosin network that allows for the “pulling” inwards of the furrows from the plasma membrane are yet unclear. Nuclei tethered to the furrows can be dragged inwards alone or along with additional nuclei, suggesting connections between nuclei may form prior to furrow invasion. **Stage 3–4, Assembly of the cellularization foam:** Tubular furrows, comprising membrane and associated actomyosin networks, branch and merge to generate the polyhedral territories of cellularization, each containing a single nucleus. The microtubules play a role in patterning the size and and geometry of the polyhedral territories. The ciliary axonemes extend in between the leaves of the double-membrane of the polyhedral faces. How the tubular furrows generate the polyhedra and how tubular structures transform into sheets is unclear. **Stage 5, not shown:** Finally, after maturation of the cellularization foam, the zoospores undergo cell separation and swim or crawl out through an opening the the cell wall of the mother.

Here, we dissect the process of chytrid 3D cellularization into molecularly distinct stages (Figure 7). Cellularization onset is marked by the migration of nuclei and their associated, outward-facing centrosomes to the plasma membrane, followed by vesicle formation at the point where the centrosome abuts the plasma membrane. These membrane structures then project inward in an actomyosin-dependent process, becoming tubular “furrows” that drag the membrane and attached nuclei into the cytoplasm. The furrows expand, branch, and merge using membrane sourced from the Golgi and eventually settle into a tessellation of tightly-packed polyhedra of homogenous volume, each containing a single nucleus. Finally, the polyhedra synchronously separate from each other to form daughter cells that crawl or swim away through a hole in the cell wall of the mother. During cellularization, one of the two centrioles associated with each nucleus transitions into a basal body and grows a cilium. We show that, although chytrid cellularization requires actomyosin networks, it uses them in ways that are drastically different from those of animals and yeast.

### Chytrids use actomyosin for cellularization but with different mechanisms from animal and yeast cytokinesis.

We use the spatial arrangement of nuclei to categorize cellularization as one-dimensional (conventional cytokinesis), two dimensional (e.g., *Drosophila* cellularization), or three-dimensional and show that chytrids use actomyosin in unique ways to drive three-dimensional cellularization. Conventional cytokinesis and *Drosophila* cellularization rely on the assembly of an actin ring at the plasma membrane that constricts and deforms the membrane into a trench-like furrow around each nucleus. Similarly, chytrids use actomyosin networks to drive membrane invagination during 3D cellularization. However, chytrid cellularization requires building furrows in regions of the cell that do not have ready access to the plasma membrane. Instead of building a furrow around each of the internal nuclei, chytrids recruit nuclei to the plasma membrane prior to furrow initiation and tether each nucleus to a furrow. Although our data lack the resolution to determine if *all* nuclei must be recruited to the plasma membrane for proper cellularization, nuclei that do migrate and tether to the plasma membrane maintain their connection to the plasma membrane while the nuclei are moved inward. The resulting tubular furrows are then used to build the cellularization tessellation. Thus, chytrid cellularization neatly solves a problem not faced by well characterized animal or yeast model systems whose nuclei are always in proximity to the plasma membrane.

Similar mechanisms to chytrid cellularization are likely deployed by other fungi that generate spores from a multinucleated sporangium, including two groups of emerging fungal pathogens, members of Mucoromycotina and *Coccidioides.* Mucoromycetes include *Mucor* and *Rhizopus* that cause the deadly black fungus disease that followed the COVID-19 pandemic (Hoenigl et al., 2022). These fungi produce spores by a mechanism of multinuclear cellularization that includes the formation of a tubular membrane network that extends inwards from the periphery to create polygonal cleavage planes around daughter nuclei (Beakes and Campos-Takaki, 1984; Beakes, 1995; Bracker, 1968; Edelmann and Klomparens, 1994; Hammill, 1981; Schwarze, 1922). Fungi of the genus *Coccidioides* (Ascomycota: Onygenales) cause the Valley fever respiratory disease (coccidioidomycosis), which is expanding due to aridification associated with climate change (Jackson et al., 2024). *Coccidioides* forms a spherical coenocyte called a spherule that undergoes multinuclear cellularization via invagination of the plasma membrane, resulting in tightly packed uninucleated polygonal endospores (Breslau et al., 1961; Cole et al., 1993; Sun et al., 1979; Sun and Huppert, 1976). Our findings suggest that chytrid multinuclear cellularization may provide a framework to understand the developmental biology of these important human pathogens.

Actomyosin-based multinuclear cellularization is not limited to fungi and *Drosophila*. The Ichtyosporeans *Sphaeroforma arctica* and *Creolimax fragrantissima* are unicellular relatives of animals that, like *Drosophila*, undergo two-dimensional multinuclear cellularization where actomyosin drives the synchronous invagination of membrane to produce an epithelia-like monolayer of cells (Dudin et al., 2019; Olivetta and Dudin, 2023; Suga and Ruiz-Trillo, 2013). Indeed, published images of cellularization in *S. arctica* under long term microtubule depolymerization treatment (Methyl Benzimidazol-2-yl-carbamate; MBC) suggest that, like chytrids, Ichtyosporeans may usemicrotubules in nuclear positioning but not cleavage (Dudin et al., 2019). The plasmodium of the acellular slime mold *Physarum polycephalum* also likely uses actomyosin for multinuclear cellularization (Goodman and Rusch, 1970; Okamura et al., 1983). Moreover, based on transitions from multinucleate to uninucleate cell states, multinuclear cellularization may also occur during the development and/or germination of slime mold sporocarps, structures that were likely present in the ancestor of the Amoebozoa (Kang et al., 2017). Because the shared ancestor of animals, fungi, and slime molds likely had a multinuclear stage (Skejo et al., 2021), it may have had the capacity to generate uninucleated propagules or gametes through multinuclear cellularization. Here we show that, like animals and Ichtyosporeans, chytrid fungi rely on actomyosin networks to drive multinuclear cellularization, suggesting that using actomyosin for this function may also be an ancestral feature of animals and fungi.

Although chytrid fungi are, to our knowledge, the first system known to use contractile actin networks for three-dimensional cellularization, they are most certainly not the only system capable of three-dimensional cellularization. This strategy is present in many eukaryotic lineages, including the distantly related land plants and Oomycetes. During cell division in land plants, a specialized structure called the phragmoplast forms a cell plate in the center of the dividing cell through polarized delivery of membrane and cell wall components This outgrowing disk eventually reaches and fuses with the plasma membrane and partitions the cell cytoplasm (Smertenko et al., 2017). Endosperm tissue of many plant species arises from the cellularization of a coenocyte through building mini-phragmoplasts in between all of the nuclei (Otegui and Staehelin, 2000; Otegui et al., 2001; Pickett-Heaps et al., 1999). Oomycetes like the plant pathogen *Phytophthora* or the fish pathogen *Saprolegnia* also produce motile zoospores via cellularization of a multinucleated sporangium (Walker and Van West, 2007). Cleavage starts by the recruitment of Golgi derived vesicles to the basal body associated with each nucleus, with centrosomal arrangements similar to what we see in chytrids. The sporangium forms a membranous cisterna around the cell periphery that then extends and merges with other membranes that extend from the nuclear centrosome to surround each individual nucleus—much like fission yeast spore formation (Hyde et al., 1991; Onishi et al., 2010). These clear differences in mechanism and strategy leave open the question whether cellularization evolved multiple times or was ancestral and has undergone rampant diversification.

### Chytrid cellularization combines ciliogenesis and cytokinetic mechanisms in a single cellularization program.

We have shown that chytrids solve the problem of surrounding non-peripheral nuclei with cleavage membranes by moving internal nuclei to the plasma membrane before redistributing them to the cytoplasm. This mechanism comprises three steps: 1) recruitment of the centriole-nuclei to the plasma membrane, 2) the formation of vesicular tubular furrows between each nuclear centrosome and the plasma membrane, and 3) the tethering of the nuclei to these furrows via the centrosome. These activities resemble key stages of animal ciliogenesis that starts with the migration of the centriole to the plasma membrane. During this migration, the distal end of one centriole recruits membrane to form a double-membrane sheet called the “ciliary vesicle”. This centriole then fuses to the plasma membrane via the ciliary vesicle and extends anaxoneme to form a cilium (Zhao et al., 2023). Chytrid vesicular furrow initials resemble animal ciliary vesicles not only in their appearance and position, but also in their genesis. Upon recruitment to the plasma membrane, chytrid nuclei interact with the vesicular furrow initials via the centrosomal region, an association that persists throughout the rest of cellularization. We therefore propose that nuclear tethering during cellularization may be equivalent to centriolar docking in animal ciliogenesis, which relies on genes associated with human ciliopathies (Tanos et al., 2013). Although many of these genes are also present in chytrids, whether chytrids rely on the same genes for ciliogenesis is an open question. Finally, ultrastructural studies of chytrid ciliogenesis show that it begins by forming a “primary flagellar vesicle” at internal basal bodies, similar to the animal “ciliary vesicle” (Barron and Hill, 1974; Lessie and Lovett, 1968; Lowry and Roberson, 1997; Renaud and Swift, 1964). The “primary flagellar vesicle” can be seen at the distal part of the basal body and against the plasma membrane during chytrid ciliogenesis (i.e. Figure 5 in Barron and Hill, 1974). We propose that this “primary flagellar vesicle” *is* the vesicular furrow initial. This would mean that ciliogenesis and cellularization in chytrids are intertwined, both spatiotemporally and mechanistically.

The similarities between animal intracellular ciliogenesis and early chytrid cellularization extend to their cytoskeletal mechanisms. We found that Nocodazole treatment did not inhibit cellularization but did inhibit axonemal elongation. This is similar to ciliogenesis in the quail oviduct, where Nocodazole treatment did not inhibit centrosome migration or centriolar docking but did inhibit axoneme elongation (Boisvieux-Ulrich et al., 1989, 1987). Similarly, actin network perturbation disrupts both chytrid cellularization and centriolar migration in the quail oviduct (Boisvieux-Ulrich et al., 1990). Thus, chytrids may use ciliogenetic mechanisms ancestral to animals and fungi to recruit the centrosomes and their nuclei to the plasma membrane. Because mechanistic details of centrosomal migration during animal ciliogenesis remain unclear (Hannaford and Rusan, 2024), the mechanisms of cortical nuclear migration in chytrids may be a fantastic model system to explore the mechanisms and the evolution of centrosomal migration and ciliogenesis in animals, fungi and their shared ancestor.

### Using chytrids to explore the physics and geometry of foams in biological systems.

We have shown that chytrids cellularize by building a tessellation of polyhedra. We found that, although mother cells vary in size and number of nuclei, the daughter zoospores they produce are remarkably uniform in size. Daughter cell size is directly linked to the geometry of the polyhedra in the tesselation; polyhedra of homogenous volume give rise to daughters of homogenous volume. So how is the pattern, size, and symmetry of the tessellation of polyhedra generated? A hint may come from the similarity of chytrid cellularization to a system of packed soap bubbles, or “foam”. A foam is a collection of surfaces that meet according to Joseph Plateau’s Laws (1873) which state that thin soap films organize in a geometry that minimizes surface area for a given volume (Delsaulx, 1884; Plateau, 1873). More than 100 years ago, the mathematician and natural philosopher D’Arcy Thompson suggested that cellular partitions, tissues, and multicellular aggregates tend to follow the geometrical configurations of soap bubbles to minimize surface area (Thompson, 1945). Since then, the soap bubble analogy has been invoked to explain foam-like patterns in biological systems that arise through distinct mechanisms, such as transmission of extracellular force across tissues, differential adhesion between cells, or space-constrained cell division (Agarwal and Zaidel-Bar, 2019; Hayashi and Carthew, 2004; Heer and Martin, 2017; Lecuit and Lenne, 2007). These processes start from separate individual “bubbles” that interact to eventually give rise to the foam. Although chytrid cellularization resembles these types of foams, the genesis of the chytrid tesselation is completely different. It does not arise from the agglomeration of separate, individual “bubbles”. Instead, all of the interfacing surfaces and individual polyhedra of chytrid cellularization foams are constructed *de novo*.

Generating zoospores is energetically expensive, and evolution may have selected for the most efficient way to compartmentalize a chytrid sporangium into zoospores of defined volume. Under this model, chytrids have evolved to solve a special case of Plateau’s problem called *Kelvin’s Problem—*finding the most efficient “tessellation of space into cells of equal volume with the least surface area” (Thomson, 1887). The solution proposed by Kelvin of a 14-sided truncated octahedron with eight slightly distorted hexagonal faces and six square faces remained unchallenged for more than 100 years until Weaire and Phelan used simulations to propose a structure that improved upon Kelvin’s by 0.3% (Weaire and Phelan, 1994). The Weaire-Phelan structure is made up of two types of cells, a 12-sided irregular polyhedron of pentagonal faces and a 14-sided polyhedron with pentagonal and hexagonal faces, with only the hexagons remaining planar. Although we lack a detailed understanding of the exact geometry of chytrid cellularization foams, we found very few instances of right angles, suggesting a structure more akin to the Weaire-Phelan than Kelvin’s. Because Kelvin’s problem must be solved by all biological systems that involve complete cellularization within a constrained volume, understanding the mechanisms used by chytrids to solve Kelvin’s problem can shed light on the principles of cellular morphogenesis across eukaryotic lineages, as well as inspire new strategies for solving geometrical problems within or without biological systems.

Applying the soap bubble analogy to chytrid cellularization can also help us think about how chytrid mothers produce homogenous daughter cells. Plateau’s laws state that: 1) the “bubbles” in the system should have smooth, unbroken surfaces, 2) all points on the same soap film surface have a constant mean curvature, 3) when soap bubble surfaces meet in threes they do so at an angle of 120 degrees—resulting in hexagonal symmetry—and 4) when they meet in fours they do so at an angle of 109.47—tetrahedral symmetry. Because chytrid cellularization involves continuous, smooth cleavage membranes as well as pentagonal- and hexagonal-faced polyhedra, and these polyhedra are mechanically connected, chytrid cellularization appears to obey at least the first three of these laws. If we assume that chytrid cellularization follows Plateau’s Laws, then chytrids cellularize under three spatial constraints: the mother’s cell volume is fixed, all compartments have the same “target” volume, and each compartment must contain a single nucleus. This raises important questions regarding the molecular mechanisms underlying how the target cell volume is defined and how the mother builds a compartment around each nucleus. Given that microtubule depolymerization results in patterning defects during chytrid cellularization, microtubules may help define proper nuclear spacing and a “minimal zoospore territory”—or “target volume”. Additionally, the tethering of nuclei to the cellularization furrows may help the mother cell keep track of all of the nuclei and serve as a guide around which to build each polyhedron.

### Concluding remarks.

Chytrid cellularization solves three-dimensional problems that neither animal nor yeast model systems face. Accordingly, we have found that chytrids fulfill each of the four fundamental steps of cellularization using mechanisms that consistently deviate from the general strategies deployed by animal and yeast model systems. First, in animal and yeast cytokinesis, nuclei find and hold their position while cleavage furrows move around them. In contrast, chytrid nuclei are highly dynamic throughout cellularization. Second, animal and yeast systems rely on plasma membrane-associated cues to recruit and assemble the cytokinetic machinery. While conceptually simple for 1D and 2D cellularization, this constraint is problematic for 3D cellularization because most nuclei lie deep in the cytoplasm, surrounded by other nuclei, and are therefore spatially separated from plasma-membrane bound cues. We found that chytrids solve this problem by recruiting and attaching the nuclei to the plasma membrane, and *then* using actomyosin to build cleavage structures away from the plasma membrane. Third, while animal and yeast cells use trench-like furrows comprising membranes and actomyosin networks as cleavage structures, chytrids use tubular furrows of membranes and actomyosin that only later assemble into a polyhedron around each nucleus. How the tubular structures of early cellularization become polyhedra and how these polyhedra separate into daughter cells remain open questions. Finally, we found that chytrid cellularization may have co-opted ciliogenesis programs, raising the question of whether the similarity between these forms of cell division reflects analogy or homology at the molecular level. Taken together, we have found that chytrids use conserved actomyosin machinery for three-dimensional cellularization but with different mechanisms from those of one- and two-dimensional cellularization—a clear reminder that conservation of machinery does not always signify conservation of mechanism.

## MATERIALS AND METHODS

### Reporter construct design, strain generation, and molecular cloning.

Constructs expressing Lifeact-mClover3v(EM112C: *H2Apr-Lifeact-mClover3-H2Ater:H2Bpr-P2A-T2A-hph-SynTer8*), Myosin light regulatory chain (MLRC) (SPPG_07268) fused to mClover3 (pEM110: *H2Apr-MLRC-mClover3-FLAG-MLRCter:H2Bpr-P2A-T2A-hph-SynTer8*), *Batrachochytrium dendrobatidis* alpha-Tubulin BDEG_00078 fused to mClover3 under the promoter of *Spizellomyces* alpha-Tubulin (SPPG_05136) (EM102: *AlphaTUBpr-mClover3-BdenAlphaTUB-BdTUBter:H2Bpr-P2A-T2A-hph-SynTer8*), *Spizellomyces* centriolar component SAS6 (SPPG_03341) fused to mClover3 (EM120: H2Apr-SAS6-mClover3-SAS6ter:H2Bpr-P2A-T2A-hph-SynTer8) were synthesized by Twist Bioscience (South San Francisco, United States) and cloned by Twist directly into the binary *Agrobacterium* plasmid pCAMBIA1300 (Addgene: 44183). Plasmids were transformed into *Spizellomyces* and transformant strains were recovered as described previously (Medina et al., 2020; Prostak et al., 2023). *Synther8* is a short synthetic terminator (No 8) from (Curran et al., 2015). Nuclear-localized mClover3 (GI3EM48; *H2Bpr-RbNLS-mClover3-ScADH1ter:H2Apr-hph-ScADH1ter*) was generated using previously published pGI3EM22C (*H2Bpr-Lifeact-tdTomato-ScADH1ter:H2Apr-hph-ScADH1ter*) (Medina et al., 2020) with Lifeact-tdTomato replaced by mClover3 with an N-terminal nuclear localization signal from *Spizellomyces* Rb protein SPPG_07796. Nuclear dynamics from Figure 2 were done with a strain carrying H2B-tdTomato from plasmid pGI3EM20C as previously described (Medina et al., 2020). We sequence-verified all constructs by whole-plasmid sequencing (Plasmidsaurus Inc.). Complete plasmid sequences and plasmids have also been deposited at Addgene.

### Preparation of semi-synchronized cultures for live cell imaging.

Two days prior to the experiment, use DS solution to harvest zoospores from an active culture of *Spizellomyces punctatus* (Koch type isolate NG-3) Barr (ATCC 48900) growing in K1 plates (50mg/L Carbenicillin and tetracycline) with or without selection (200mg/L Hygromycin) as done previously (Medina et al., 2020) and filter zoospores through sterile 40-μm mesh filter (fisherbrand Cat. No. 22363547) followed by a sterile Whatman No1 syringe filter (~11 μm). Plate 1mL of 1/5–1/10 dilution in DS solution of the zoospores into new K1 plates (right out of the fridge), seal with parafilm, and incubate for ~20–24h at 28 Celsius until peak zoospore release—zoospores are ready for seeding the imaging plates.

To prepare the imaging plates, a cellvis 6-well glass bottom black plate with lid (P06–1.5H-N) is cleaned with a plasma cleaner for four minutes at maximum level (Harrick Plasma Expanded Plasma Cleaner 115V). Add promptly 1mL per well of 25ug/mL filter-sterilized 50% w/v polyethyleneimine (PEI) (sigma-Aldrich P3143–100ML) and incubate at room temperature for 10 min. After the 10 min, remove the PEI solution and rinse 10 times with 2mL of sterile DS solution, then add 2mL of sterile DS in preparation for inoculation. Do not let the treated surface dry out. Proceed to prepare the zoospore inoculum.

To seed the imaging plate, harvest and filter the zoospores from the semi-synchronous plate culture made the day before using the same approach as above. Quantify zoospore concentration with a hemocytometer by counting 10uL from a 30uL 1:10 zoospore dilution including 3uL of Lugol solution (Sigma-Aldrich; 62650–100ML-F) (killing zoospores and increasing contrast). Dilute the zoospores with sterile DS to achieve a working stock of 1×10^6 zoospores per mL. Add 1mL of 1×10^6 zoospores per mL of zoospore solution to each well and leave for 10 min without moving at room temperature. After 10 minutes, tilt and remove the excess zoospore solution and replace it with 2mL of pre-warmed 28C K1 liquid media (50mg/mL carbenicillin; Fisher AAJ6194906), place on top of a 28C pre-warmed aluminum heatblock (two small tube heatblocks taped together) and leave for 10 min. After 10 min, wash gently three times with 2mL of liquid K1 (50mg/mL carbenicillin), then fill with 3mL of liquid K1 (50mg/mL carbenicillin), seal the plate with parafilm and incubate inside a closed plastic container with a humidity chamber (empty pipette tip box with perforated tip rack filled with 50mL water). Incubate for ~18h, at which point they are ready to start cellularization.

### Live cell microscopy and laser ablation.

For live cell imaging, cells were imaged on a Nikon Ti2 microscope equipped with a Crest X-Light V2 L-FOV spinning disk (50 μm pinhole), a Prime95B sCMOS camera (Photometrics), and a Plan Apo λ 100× 1.45 NA oil objective, and an Okolab stage top chamber (H301-K-FRAME) with humidity control. The illumination setup was a Celesta Light Engine solid-state laser launch (Lumencor), Celesta VCGRnIR pentaband emitter 10–10857, and Celesta VCGRnIR penta-band dichroic 10–10858. mClover3 Emission filter ET535/70m (Chroma), FM4–64 and tdTomato emission filter ET610/75m emission. The microscope was controlled through NIS-Elements software (Nikon).

Data for figures 1A and F imaging plates were prepared as described above, but before imaging a small 1.5% low-melt agarose (Sigma, A9045) pad made in K1 media was placed on top of the cells before imaging. Images were acquired using a Leica Stellaris 8 STED mounted on a Leica DMI8-CS base and using an HC PL APO CS2 86x/1.20 water objective, and Power HyD spectral detector. Images were acquired with White Light Laser (WLL) at 0.85 power percentage, taken at 1.31X zoom, 16x line averaging, 1024×1024 resolution, and mClover target wavelengths of 509.62–586.36nm and FM4–64 target wavelengths of 624.50–771.73nm. For three-dimensional reconstruction in Figure 1A, images were acquired at 1024×1024×130 with voxel size of 0.0254340084638276 × 0.0254340084638276 × 0.14839496124031007 μm and visualized in ChimeraX-1.61(Goddard et al., 2018; Pettersen et al., 2021) with gaussian smoothing of nuclei and membrane (sdev 0.05).

For laser ablation of F-actin structures during cellularization (Figure 6), imaging plates of a strain expressing Lifeact-mClover3 were prepared as described for live cell imaging but with a small (2mL) pad of 10% gelatin (Fisher Scientific G7–500) made in K1 media placed on top of the center of the well just before imaging. Imaging was done in a Nikon Ti-E microscope equipped with an Andor Dragonfly spinning disk confocal fluorescence microscope equipped with 100x NA 1.45 objective (Nikon) and a built-in 1.5x magnifier; 488 nm diode laser with Borealis attachment (Andor); emission filter Chroma ET525/50m; and an EMCCD camera (iXon3, Andor Technology). Fusion software (Andor) was used to control data acquisition.

Ablations were performed using a MicroPoint (Andor) system with galvo-controlled steering to deliver 20–30 3ns pulses of 551 nm light at 16–20 Hz (Andor) mounted on the Dragonfly microscope described above, as previously described (Moshtohry et al., 2022; Zareiesfandabadi and Elting, 2022). Fusion software (Andor) was used to control acquisition while IQ software (Andor) was used simultaneously to control laser ablation. At this pulse rate, the ablation process lasts ~2 s. Chroma ET610LP mounted in the dichroic position of a Nikon filter turret was used to deliver the ablation laser to the sample. Since this filter also reflects the mEGFP emission, the camera frames collected during the ablation process are blank. The behavior of the severed F-actin structures of cellularization was imaged before and immediately following laser ablation by acquiring a single confocal plane in the 488-nm channel every second for at least one minute. The mechanism of ablative photodecomposition remains unclear but may be caused by either the propagation of a pressure wave and/or cavitation bubble dynamics (Rau et al., 2006; Venugopalan et al., 2002). The size of the damage is approximately the size of the diffraction spot of the lens <0.4 μm in the XY plane and <0.8 μm in the Z axis (Khodjakov et al., 1997).

### Pharmacological perturbations during live-cell imaging.

About one hour before the start of the experiment (~17h), remove excess of dislodged sporangia by removing gently 2mL of the media and replacing it with 2mL of fresh prewarmed K1+Carb media, adding and removing liquid from opposite sides of the well. Repeat this wash thrice in total. To the remaining 1mL, add 1mL of 20uM FM4–64 (stock 1mg/mL in water) made in K1+Carb media for staining the plasma membrane. Bring to the microscope and place in a preheated Okolab stage top chamber (H301-K-FRAME) with humidity control (30 Celsius). Twenty individual sporangia were selected from a single well for time-lapse imaging. Imaging started at 18h, sampling every 3 or 5 min, for six hours. Pharmacological perturbations are applied by removing 1mL of media and replacing it with 2X working solution of the drug of choice. For washout experiments, 50% of the final volume of the well is replaced with fresh pre-heated media ten times. Cells were treated with 2uM final concentration of Nocodazole (Cayman Chemical; No. 13857) or equivalent volume of DMSO, 50uM of Brefeldin A (Sigma B7651) or equivalent volume of DMSO, 10uM Latrunculin B (Millipore, 428020) or equivalent volume of ethanol, 150uM p-a-blebbistatin (Cayman 22699) or equivalent volume of DMSO.

### Coulter Counter cell size measurements.

1mL of zoospores from six independent culture plates of *Spizellomyces* were freshly harvested as described in the section on semi-synchronized cultures for live imaging was placed in a 25mL Accuvette (A35473; Beckman Coulter) with 24mL of freshly made 50% dilution of Isoton II diluent (C96980; Beckman Coulter) and run through a 10uM aperture tube (B42812; Beckman Coulter) on a Coulter Counter Multisizer 4e (B23005; Beckman Coulter) running on a freshly made 50% dilution of Isoton II diluent (C96980; Beckman Coulter). The Coulter counter is calibrated with 2-μm latex beads (C72513 CC; Beckman Coulter). The size distribution of zoospores and beads was exported as CSV, then processed, and visualized using JupyterLab 1.16 and R v4.1.2 facilitated by custom scripts (Kluyver et al., 2016; R Core Team, 2013).

### Image processing and analyses.

To measure the angle distribution in the polyhedral tessellation of cellularization (Figure 1E), five sporangia from three independent biological replicates of control cells stained with FM4–64 and imaged during cellularization were selected. The frame before obvious signs of cellularization was selected for each sporangium and the angles between the three cleavage planes (tri-furrow) around at least ten different nodes (where furrows meet) were measured using the angle function of Fiji (Schindelin et al., 2012). As a positive control and a gauge of technical reproducibility, an artificially perfect 120-degree tri-furrow with similar proportions of thickness-length was used for generating an artificial dataset by transposition and measured the same way at the same time. The resulting dataset was processed and visualized using JupyterLab 1.16 and R v4.1.2 facilitated by custom scripts (Kluyver et al., 2016; R Core Team, 2013).

For measuring nuclear cortical migration dynamics (Figure 2B) we used scikit-image (Van der Walt et al., 2014) image processing in Python with custom scripts in a Google Collab notebook. Briefly, elliptical sporangia were masked, and segmented and an ellipse was fitted to their perimeter. A second ellipse with 70% of radii from the perimeter ellipse was used as the boundary between the outer and inner 50% of the sporangial area. The nuclei were segmented via thresholding and watershed algorithms. The cortical nuclear ratio is the number of nuclei in the outer donut relative to the inner disk for each frame in the time-lapse video. The resulting measures were then processed and visualized using JupyterLab 1.16 and R v4.1.2 facilitated by custom scripts (Kluyver et al., 2016; R Core Team, 2013).

To measure the defect in patterning observed after Nocodazole treatment in cellularizing sporangia (Figure 4A & B), an ellipse was masked and fitted to circular sporangia as described above using scikit-image (Van der Walt et al., 2014) image processing in Python with custom scripts in a Google Collab notebook. Briefly, since the cleavage furrows of the outer layer of daughter cells are perpendicular to the perimeter cell wall, and thus size estimates are likely to be more accurate, a concentric ellipse at 0.85% of the fitted perimeter ellipse was drawn. The peak-to-peak distance along the profile of the FM4–64 fluorescence intensity along the perimeter of this ellipse was used as a proxy for daughter cell diameter. The resulting measures were then processed and visualized using JupyterLab 1.16 and R v4.1.2 facilitated by custom scripts (Kluyver et al., 2016; R Core Team, 2013). The statistical significance test was a one-tailed permutation test of the difference of means.

The effects of Brefeldin A on cellularization were visualized by similar sporangial segmentation and masking followed by log-polar transform using sci-kit-image (Van der Walt et al., 2014) image processing in Python with custom scripts in a Google Collab notebook. The phenotyping of the sporangia (success, Failure to Start, Network Collapse) was done by hand in Fiji (Schindelin et al., 2012). The resulting measures were then processed and visualized using JupyterLab 1.16 and R v4.1.2 facilitated by custom scripts (Kluyver et al., 2016; R Core Team, 2013).

To measure the effects of Latrunculin B on the F-actin structures during cellularization, sporangia of circular shape that did not move excessively during the experiment were selected and imported to CellProfiler 4.2.1 (McQuin et al., 2018) for segmentation. As a proxy of the level of cellularization in the actin structures, we used Haralick measures of texture (Haralick et al., 1973), the Angular Second Moment relative to the time-point right before LatB treatment—relative homogeneity—was used for visualization. The phenotyping of the effects of p-a-Blebbistatin on cellularization (completion or failure) was done by hand in Fiji (Schindelin et al., 2012).

For Particle Image Velocimetry (PIV) analyses, selected sporangia were masked in Fiji (Schindelin et al., 2012) and analyzed with OpenPIV-0.25.0 (Liberzon et al., 2021) through custom Python scripts in a Google Collab Notebook. Vector field plots (Figure 6B) and plots from the top 10% velocity vectors per PIV timepoint (Figure 6C) were generated in JupyterLab 1.16 and R v4.1.2 facilitated by custom scripts (Kluyver et al., 2016; R Core Team, 2013). Kymographs from Figure 6A were generated using Fiji (Schindelin et al., 2012) from regions 20-pixel wide centered and parallel to the ablated furrow. Recoil displacement analyses from laser ablation of late-stage polyhedra were performed via sci-kit-image (Van der Walt et al., 2014) image processing in Python with custom scripts in a Google Collab notebook. Briefly, a 10-pixel wide region centered in a line along the ablated furrow was used to measure the mean fluorescence intensity profile along the furrow. Intensity peaks were detected and used to locate and measure the inter-peak distance between the receding tips of the ablated furrow. The results were visualized using JupyterLab 1.16 and R v4.1.2 facilitated by custom scripts (Kluyver et al., 2016; R Core Team, 2013).

A main concern during our analysis was the difference in time between image acquisition before and after ablation and the time required for ablation. PIV uses the displacement of tracer particles (pixels showing fluorescence of F-actin in our case) between consecutive images to estimate the changes in direction and velocity of the tracer particles. Although we imaged every second before and after laser ablation, it takes about two seconds to perform the 20–30 pulses of the laser required to sever the actin structures and one more second to remove filters and take the next image. Thus, our images before and after ablation are separated by three seconds, while in reality, there is about one second from the end of the laser ablation until the acquisition of the first image “post” ablation. Furthermore, we do not have the “real” image of the sporangium just before ablation, only before ablation is started. To navigate this problem we could assume that the sporangium before the start of ablation is a good proxy of the status of the actin structures before the end of ablation, and thus run our PIV analysis using a sampling time of one second before and after ablation. Alternatively, we can run the PIV analysis using three seconds as the sampling time between images, which would underestimate by up to three-fold the velocities in response to ablation, but it would be accurate about the true time difference between images. A second concern is that when all pixels within sporangium are used as tracer particles, we would detect displacement not only in cleavage structures but also movements of the fluorescent probe in the cytoplasm.

We tested different strategies of resampling and filtering the ablation data of a sporangium to assess their effects on the recoil signal retrieved by PIV Figure S3 & S4. We performed basic thresholding (MultiOtsu) to remove the cytoplasmatic signal, resampled the complete time-series every three seconds to reflect the true time difference between pre-ablation and post-ablation images, combined thresholding and resampling, and combined resampling with a mask (generated from the sum of all frames before ablation followed by MultiOtsu thresholding) to restrict the PIV to the cleavage structures. To measure the displacement caused by ablation, we retrieved vectors with magnitude greater than zero and that pass PIV signal to noise ratio test and calculated their radial component of the velocity (V) relative to the ablation site (𝒜) (Figure 6C **cartoon**). This measure $\left({\text{V}}_{𝒜}\right)$ represents, for a velocity vector anywhere in the sporangium, the part of the velocity vector that is in the direction of the ablation site 𝒜. This allows us to probe specifically the displacement velocities away $\left({\text{V}}_{𝒜}>0\right)$ or towards $\left({\text{V}}_{𝒜}<0\right)$ the ablation site irrespective of the complexity of geometry and movement of the actin structures across the sporangium. We found that the recoil signal is robust to different strategies of data processing, thus we decided to use three-second resampling of the dataset without thresholding as the main data processing for our analyses and discussion.

Quantification of recoil displacement after ablation of the structures of late cellularization (Figure 6E–F) performed by calculating the distance between severed furrow tips. Tip position defined as the peak in the fluorescence intensity distribution in a line 10-pixel wide along the ablated cleavage plane (white dotted rectangle in inset).

## Supplementary Material

Supplement 1 Supplementary Video 1. Distribution of nuclei during chytrid stage 4 peak cellularization. Nuclei (RbNLS-mClover3) shown in green and membrane (FM4-64) in magenta. Laser-scanning confocal imaging. Mother cell is about 20 μmmicrons deep: Z-plane sweep of 130 Z-stacks, each 0.148 μm. Scale 5 μm.

Supplement 2 Supplementary Video 2. F-actin colocalizes with the cellularization membrane polyehedra of a live chytrid mother cell during stage 4 about to reach peak cellularization. F-actin (Lifeact-mClover3) shown in green and membrane (FM4-64) in magenta. Note a small section is not completely cellularized. Laser-scanning confocal imaging. Mother cell is about 20 μm deep: Z-plane sweep of 138 Z-stacks, each 0.148 μmmicrons. Scale 5 μm.

Supplement 3 Supplementary Video 3. Distribution of myosin-II light regulatory chain (MLRC) during chytrid peak cellularization. A probe of myosin-II activity, MLRC (MLRC-mClover3) in shown in green and membrane (FM4-64) in magenta. Laser-scanning confocal imaging. Mother cell is about 20 μm deep: Z-plane sweep of 136 Z-stacks, each 0.148 μm. Scale 5 μm.

Supplement 4 Supplementary Video 4. Microtubule architecture during chytrid Stage 4, peak cellularization. Chytrid alpha-tubulin (mClover3-alpha-Tubulin) in shown in green and membrane (FM4-64) in magenta. Laser-scanning confocal imaging. Z-plane sweep of 28 Z-stacks, each 0.6 μm. Scale 5 μm.

Supplement 5 Supplementary Video 5. Nuclear dynamics during chytrid cellularization. Nuclei visualized with fluorescently-tagged histone H2B (H2B-tdTomato) are shown in green and membrane (FM4-64) in magenta. Spinning-disk confocal time-lapse. Sampling rate of one image per minute. You can see the last two nuclear divisions before the chytrid mother enters cellularization. Scale 5 μm.

Supplement 6 Supplementary Video 6. Example of Nuclear cortical migration during chytrid cellularization. Nuclei visualized with fluorescently-tagged histone H2B (H2B-tdTomato) are shown in green and membrane (FM4-64) in magenta. Nuclei reach peak cortical ratio around 295 minutes. Spinning-disk confocal time-lapse. Sampling rate of one image per minute. You can see the last two nuclear divisions before the chytrid mother enters cellularization. Scale 5 μm.

Supplement 7 Supplementary Video 7. Example 1 of outward-facing nuclear centrosomes during entry into cellularization. Fluorescently-tagged tubulin (mClover3-alpha-Tubulin) shown in green and membrane (FM4-64) in magenta. Note how during cortical nuclear recruitment, the nuclear-associated centrosomes are often positioned towards the plasma membrane (100-110 min). Spinning-disk confocal time-lapse. Sampling rate of one image every five minutes. Scale 5 μm.

Supplement 8 Supplementary Video 8. Example 2 of outward-facing nuclear centrosomes during entry into cellularization. Fluorescently-tagged tubulin (mClover3-alpha-Tubulin) shown in green and membrane (FM4-64) in magenta. Mitotic spindles are visible during nuclear division (85min), and during entry into cellularization (140 min). Note how during cortical nuclear recruitment, the nuclear-associated centrosomes are often positioned towards the plasma membrane (135 min). Nuclei remain tethered to the invading furrow via the centrosome. Spinning-disk confocal time-lapse. Sampling rate of one image every five minutes. Scale 5 μm.

Supplement 9 Supplementary Video 9. Example 3 of outward-facing nuclear centrosomes during entry into cellularization. Fluorescently-tagged tubulin (mClover3-alpha-Tubulin) shown in green and membrane (FM4-64) in magenta. Note how during cortical nuclear recruitment, the nuclear-associated centrosomes are often positioned towards the plasma membrane (25 min). Nuclei remain tethered to the invading furrow via the centrosome. Spinning-disk confocal time-lapse. Sampling rate of one image every five minutes. Scale 5 μm.

Supplement 10 Supplementary Video 10. Example 4 of microtubular dynamics during chytrid cellularization. Fluorescently-tagged tubulin (mClover3-alpha-Tubulin) shown in green and membrane (FM4-64) in magenta. Spinning-disk confocal time-lapse. Sampling rate of one image every five minutes. Scale 5 μm.

Supplement 11 Supplementary Video 11. Chytrid centrioles appear to dock and tether to the cellularization furrows during chytrid cellularization (Example 1). Fluorescently-tagged component of the centriole SAS6 (SAS6-mClover3) shown in green and membrane (FM4-64) in magenta.Spinning-disk confocal time-lapse. Sampling rate of one image every three minutes. Scale 5 μm.

Supplement 12 Supplementary Video 12. Chytrid centrioles appear to dock and tether to the cellularization furrows during chytrid cellularization (Example 2). Fluorescently-tagged component of the centriole SAS6 (SAS6-mClover3) shown in green and membrane (FM4-64) in magenta. Spinning-disk confocal time-lapse. Sampling rate of one image every three minutes. Scale 5 μm.

Supplement 13 Supplementary Video 13. Chytrid mothers exposed to Brefeldin-A before entry into cellularization fail to enter cellularization. Fluorescently-tagged tubulin (mClover3-alpha-Tubulin) shown in green and membrane (FM4-64) in magenta. Spinning-disk confocal time-lapse. Sampling rate of one image every three minutes. Scale 5 μm.

Supplement 14 Supplementary Video 14. Chytrid mothers exposed to Brefeldin-A during late cellularization show collapse of the cellularization furrows. Fluorescently-tagged tubulin (mClover3-alpha-Tubulin) shown in green and membrane (FM4-64) in magenta. Spinning-disk confocal time-lapse. Sampling rate of one image every three minutes. Scale 5 μmmicrons.

Supplement 15 Supplementary Video 15. The actin-based furrows of early cellularization are under tension and recoil where severed by laser ablation (Example 1). Spinning-disk confocal time-lapse imaging of central plane of a strain expressing the F-actin probe Lifeact-mClover. The actin structures are severed using a laser. Note the fast recoil response after ablation. Scale 5 μm.

Supplement 16 Supplementary Video 16. The actin-based furrows of early cellularization are under tension and recoil where severed by laser ablation (Example 2). Spinning-disk confocal time-lapse imaging of central plane of a strain expressing the F-actin probe Lifeact-mClover. The actin structures are severed using a laser. Note the fast recoil response after ablation. Scale 5 μm.

Supplement 17 Supplementary Video 17. The actin-based furrows of early cellularization are under tension and recoil where severed by laser ablation (Example 3). Spinning-disk confocal time-lapse imaging of central plane of a strain expressing the F-actin probe Lifeact-mClover. The actin structures are severed using a laser. Note the fast recoil response after ablation. Scale 5 μm.

Supplement 18 Supplementary Video 18. The actin-based furrows of early cellularization are under tension and recoil where severed by laser ablation (Example 4). Spinning-disk confocal time-lapse imaging of central plane of a strain expressing the F-actin probe Lifeact-mClover. The actin structures are severed using a laser. Note the fast recoil response after ablation. Scale 5 μm.

Supplement 19 Supplementary Video 19. The actin-based furrows of early cellularization are under tension and recoil where severed by laser ablation (Example 6). Spinning-disk confocal time-lapse imaging of central plane of a strain expressing the F-actin probe Lifeact-mClover. The actin structures are severed using a laser. Note the fast recoil response after ablation. Scale 5 μm.

Supplement 20 Supplementary Video 20. The actin-based furrows of early cellularization are under tension and recoil where severed by laser ablation (Example 7). Spinning-disk confocal time-lapse imaging of central plane of a strain expressing the F-actin probe Lifeact-mClover. The actin structures are severed using a laser. Note the fast recoil response after ablation. Scale 5 μm.

Supplement 21 Supplementary Video 21. The polyhedra of chytrid cellularization are under tension (Example 1). Spinning-disk confocal time-lapse imaging of central plane of a strain expressing the F-actin probe Lifeact-mClover. The actin structures are severed using a laser. Note the fast recoil response after ablation followed by deformation of the neighboring polyhedra. Scale 5 μm.

Supplement 22 Supplementary Video 22. The polyhedra of chytrid cellularization are under tension (Example 2). Spinning-disk confocal time-lapse imaging of central plane of a strain expressing the F-actin probe Lifeact-mClover. The actin structures are severed using a laser. Note the fast recoil response after ablation followed by deformation of the neighboring polyhedra. Scale 5 μm.

Supplement 23 Supplementary Video 23. Effects of Nocodazole on microtubule and membrane structures during cellularization. Data shown here is an expanded field of view associated with Figure S2.

Supplement 24 Supplementary Video 24. Live cell imaging at higher frequency (1/30 sec) of centrosomal dynamics (SAS6-mClovery) during entry into cellularization. Note that the centrosomes appear to reach the plasma membrane before the vesicular furrows are visible (Stage 1). Imaging at this frequency likely results in phototoxicity with effects that appear visible after 30 minutes of imaging (halt of centrosomal dynamics) and result in eventual death (widespread FM4-64 signal and complete arrest in internal dynamics).

Supplement 25 Supplementary Video 25. Live cell imaging at higher frequency (1/30 sec) of centrosomal dynamics (SAS6-mClovery) showing tethering of nuclear-associated centrosomes to the cellularization furrows. Note how the centrosomes follow, track, and move along the cellularization furrows.

Supplement 26 Supplementary Video 26. Live cell imaging at higher frequency (1/30 sec) of centrosomal dynamics (SAS6-mClovery) during entry into cellularization in the presence of ascorbic acid. Note that the centrosomes appear to reach the plasma membrane before the vesicular furrows are visible (Stage 1). We were able to extend our imaging times to around one hour with the aid of 500 μM ascorbic acid as a reactive oxygen species scavenger but found likely phototoxicity effects beyond that time. Ascorbic acid also caused increased background fluorescence in the mClover3 channel likely due to pH side effects on FM4-64.

Supplement 27 Supplementary Video 27. Furrows of sporangia in very early cellularization are under tension because they recoil when ablated. Spinning-disk confocal time-lapse imaging of central plane of a strain expressing the F-actin probe Lifeact-mClover during very early cellularization. The actin structures are severed using a laser. Note the fast recoil response after ablation. Scale 5 μm.

Supplement 28
