# Supplementary material for: Three-dimensional cellularization in chytrid fungi uses distinct mechanisms from those driving one- and two-dimensional cytokinesis in animals and yeast
Source: bioRxiv. 2025 Sep 24:2025.01.30.635136. Preprint. [Version 3] doi: 10.1101/2025.01.30.635136 (PMC11974727; doi:10.1101/2025.01.30.635136)
Supplement: Supplementary file 1 [file NIHPP2025.01.30.635136v3-supplement-28.pdf]

bioRxiv preprint doi: https://doi.org/10.1101/2025.01.30.635136; this version posted September 24, 2025. The copyright holder for this preprint (which was not certified by peer review) is the author/funder, who has granted bioRxiv a license to display the preprint in perpetuity. It is made available under aCC-BY 4.0 International license.

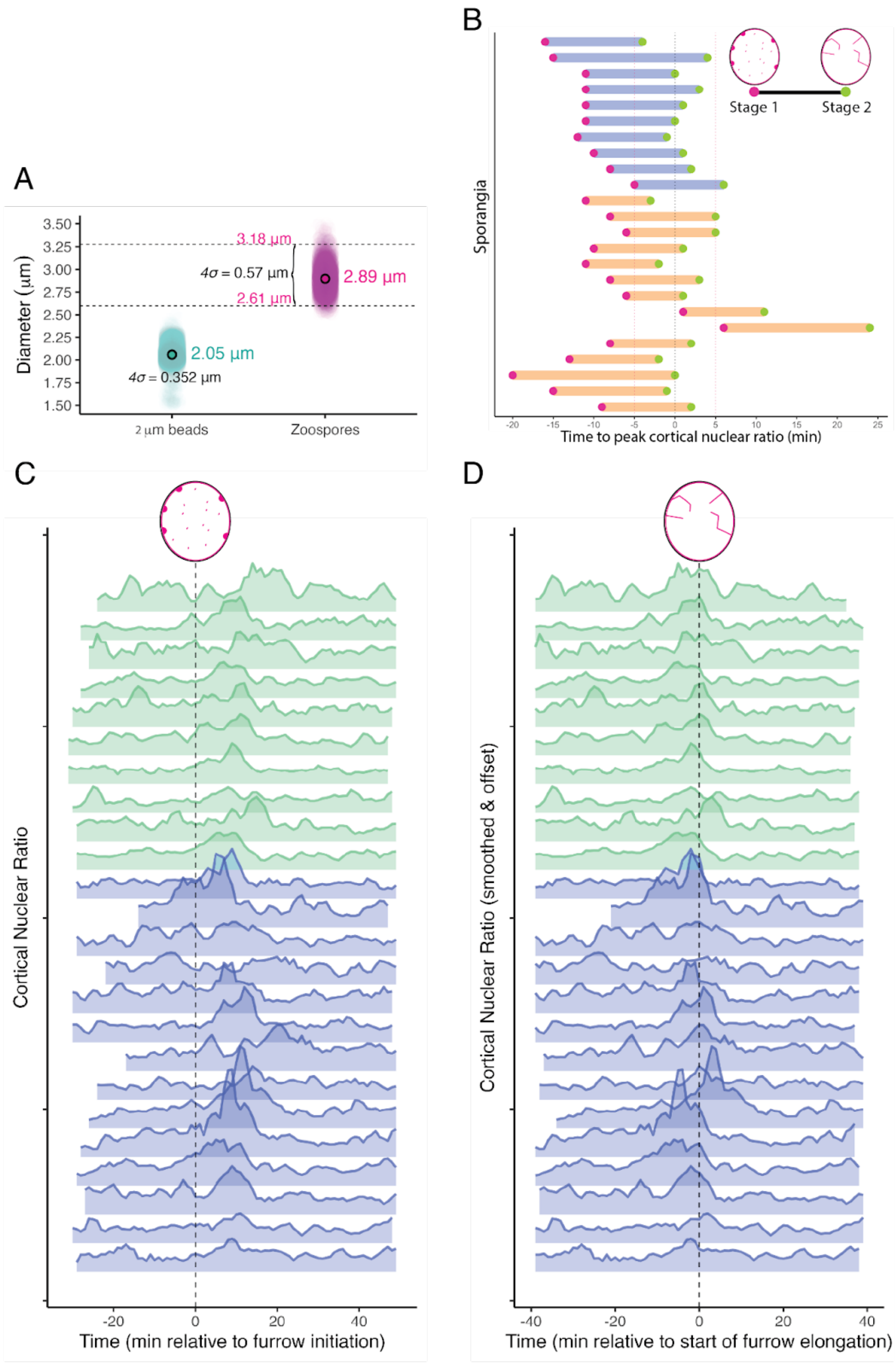

1426

1427 **Figure S1. Chytrid cellularization start coincides with recruitment of nuclei to the plasma**
1428 **membrane and produces daughter cells of homogenous size. (A)** Distribution of sizes of

bioRxiv preprint doi: https://doi.org/10.1101/2025.01.30.635136; this version posted September 24, 2025. The copyright holder for this preprint (which was not certified by peer review) is the author/funder, who has granted bioRxiv a license to display the preprint in perpetuity. It is made available under aCC-BY 4.0 International license.

chytrid zoospores (magenta) relative to artificial latex beads of 2 µm (teal). 95% of zoospores are within 0.57 µm of the mean (2.89 µm). $N_{zoospores}$ = 3785, N_{beads}= 7272. Measurements made with a Coulter counter. (**B)** Timing and duration from Stage 1 (magenta; vesicular furrow formation) to Stage 2 (green; furrow elongation) for each sporangium (row) relative to peak cortical nuclear migration . Different bar colors represent experiments from different days. (**C & D)** Cortical nuclear ratio (y-axis) relative to Stage 1 (C) or relative to Stage 2 (D). Note how peak ratio tends to happen after Stage 1 and coincides with Stage 2. Same data as in A. Different bar colors represent experiments from different days.

bioRxiv preprint doi: https://doi.org/10.1101/2025.01.30.635136; this version posted September 24, 2025. The copyright holder for this preprint (which was not certified by peer review) is the author/funder, who has granted bioRxiv a license to display the preprint in perpetuity. It is made available under aCC-BY 4.0 International license.

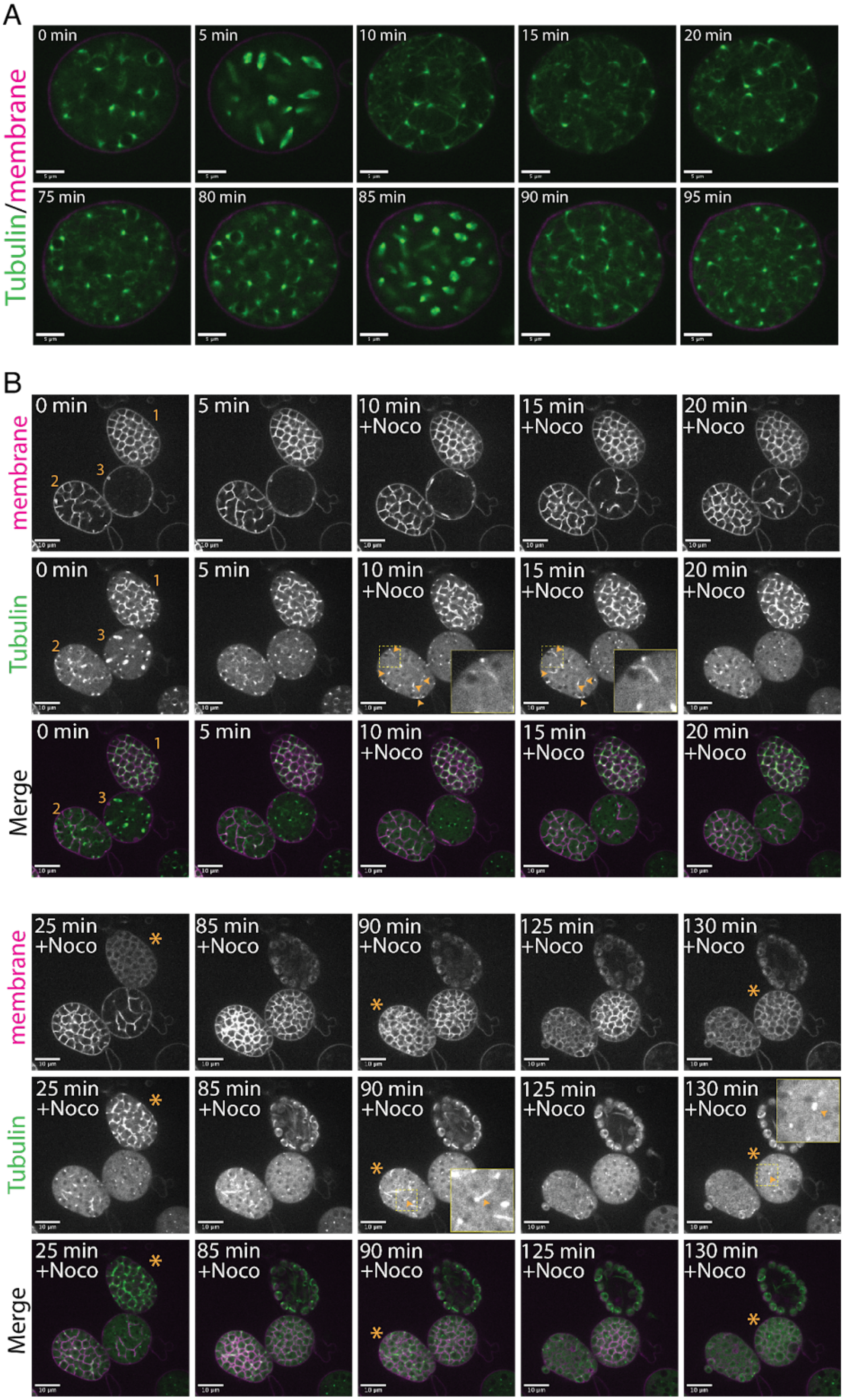

1438

bioRxiv preprint doi: https://doi.org/10.1101/2025.01.30.635136; this version posted September 24, 2025. The copyright holder for this preprint (which was not certified by peer review) is the author/funder, who has granted bioRxiv a license to display the preprint in perpetuity. It is made available under aCC-BY 4.0 International license.

Figure S2. Chytrid microtubule structures highlighted by the fluorescently-tagged alpha-tubulin and the effects of Nocodazole in these structures and ciliogenesis during cellularization.(A) Nuclear-associated foci of fluorescently-tagged tubulin are likely chytrid centrosomes. Time-series Images shown of a sporangium during growth, before entering into cellularization. Expression of fluorescently-tagged chytrid alpha-tubulin highlights centrosome-like foci at the poles of each nuclei that turn into opposing spindle poles during nuclear division. Scale bar is 5 µm. **(B)** Nocodazole does not inhibit cellularization but produces defects in patterning and aciliated daughters. Example of three mother cells at different stages in cellularization during treatment with 2 µM Nocodazole (10 min onwards). Note how Nocodazole causes disipation of fluorescent structures except for the bright foci at each nuclei (putative centrosome) and defects in polyhedral patterning. Mother 1 (see yellow numbers) is fully cellularized (Stage 4) with daughters fully ciliated. This mother completes cellularization (25 min; asterisk) and daughters escape the mother's cell wall. Mother 2 is at stage 2 of cellularization (branching & merging) and shows some nuclei starting to extend their axoneme (yellow arrows; 10 min) by the time of treatment. This results in a combination of daughters with and without cilia, but still completes cellularization (90 min; asterisk). Mother 3 is at stage 1-2 of cellularization (furrow initiation). Cellularization is completed at 130 min (asterisk) and produces daughter that completely lack cilium. Scale bar 10 µm.

bioRxiv preprint doi: https://doi.org/10.1101/2025.01.30.635136; this version posted September 24, 2025. The copyright holder for this preprint (which was not certified by peer review) is the author/funder, who has granted bioRxiv a license to display the preprint in perpetuity. It is made available under aCC-BY 4.0 International license.

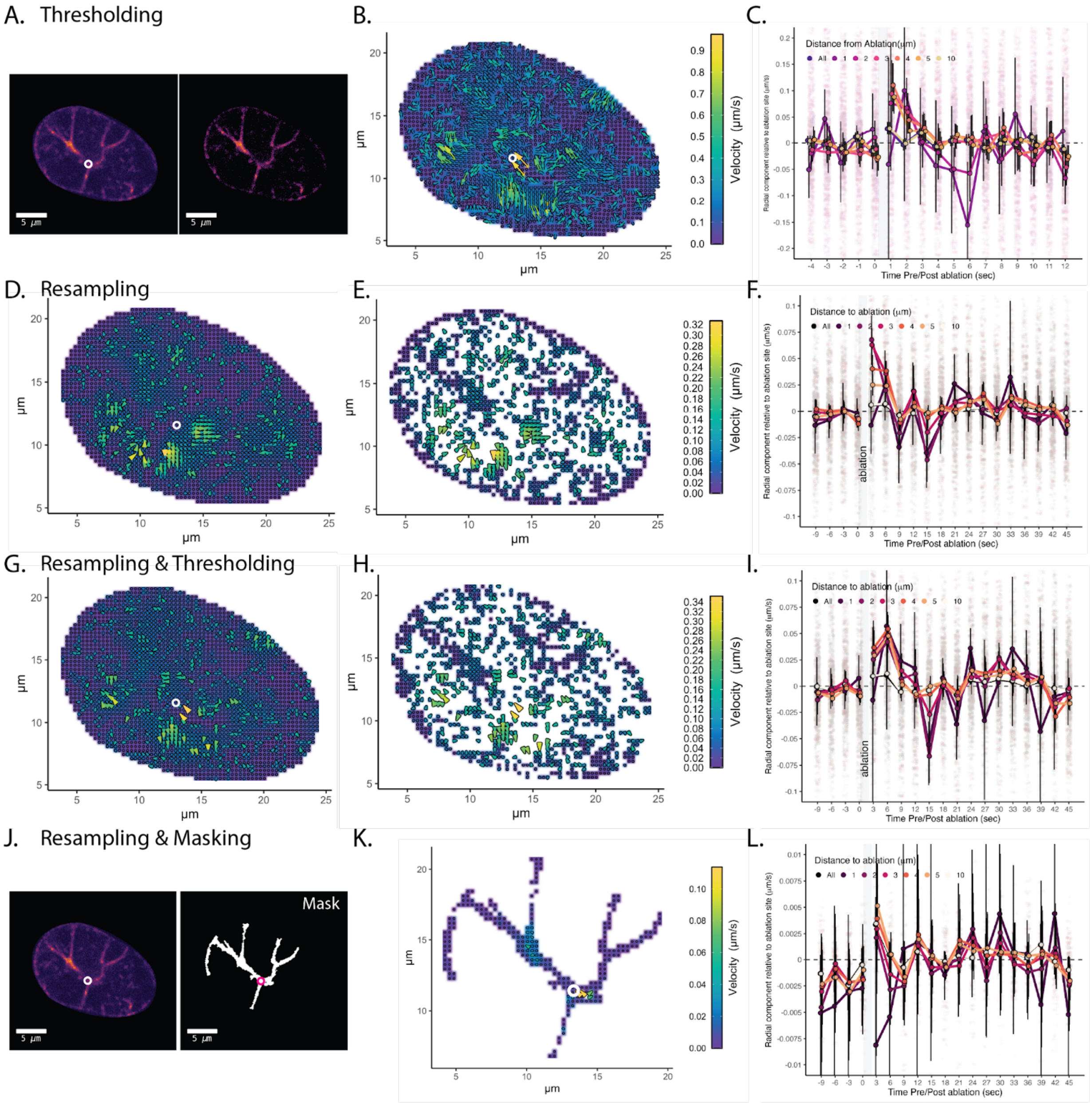

Figure S3.The signal of recoil after ablation from PIV analysis is robust to different ways of filtering and thresholding the data. (A). Example images before and after thresholding with Multi-Otsu. **(B)** Flow plot with local velocities estimated by PIV comparing the frame just before ablation (time 0) and the first frame after laser ablation (1 second from the end of ablation)—real time between images is 3 seconds, but time after the end of ablation is about 1 second. **(C)** Plot showing the radial component of velocities relative to the ablation site of all vectors vectors with magnitude above zero and that pass signal to noise test ('peak2peak'; threshold=0.5) from OpenPIV. Dots are mean values and error bars show 99% confidence interval of the mean by bootstrap. For visualization purposes only data within -0.2 and 0.2 μm/s is shown (42573 points; 85.6% of data). **(D)** Flow plot with local velocities estimated by PIV after resampling the image data every 3 seconds such that the complete time series reflect the same sampling rate as before-after laser ablation. **(E)**. Vectors from PIV that pass OpenPIV signal to noise test ('peak2peak'; threshold=0.5) and have a magnitude greater than zero are the ones used for

bioRxiv preprint doi: https://doi.org/10.1101/2025.01.30.635136; this version posted September 24, 2025. The copyright holder for this preprint (which was not certified by peer review) is the author/funder, who has granted bioRxiv a license to display the preprint in perpetuity. It is made available under aCC-BY 4.0 International license.

estimating the radial component of velocities relative to the ablation site. **(F)** Plot showing the radial component of velocities relative to the ablation site for the resampled images. Dots are mean values and error bars show 99% confidence interval of the mean by bootstrap. For visualization purposes only data within -0.2 and 0.2 µm/s is shown (537754 points; 95% of data). **(G)** Flow plot with local velocities estimated by PIV after a combination of resampling and thresholding. **(H)** Vectors from (G) that pass signal to noise and have magnitudes greater than zero. **(I)** Plot showing the radial component of velocities relative to the ablation site for the resampled and thresholded images. Dots are mean values and error bars show 99% confidence interval of the mean by bootstrap. For visualization purposes only data within -0.2 and 0.2 µm/s is shown (49370 points; 92% of data). **(J)** Masking the resampled images to limit the PIV analyses to the furrow regions (based on thresholding of the sum of the images before ablation). **(K)** Flow plot with local velocities estimated by PIV comparing the frame just before ablation (time 0) and the first frame after laser ablation (3 seconds). **(L)** Plot showing the radial component of velocities relative to the ablation site for the resampled and masked images. Dots are mean values and error bars show 99% confidence interval of the mean by bootstrap. For visualization purposes only data within -0.01 and 0.01 µm/s is shown (12042 points; 92% of data).

bioRxiv preprint doi: https://doi.org/10.1101/2025.01.30.635136; this version posted September 24, 2025. The copyright holder for this preprint (which was not certified by peer review) is the author/funder, who has granted bioRxiv a license to display the preprint in perpetuity. It is made available under aCC-BY 4.0 International license.

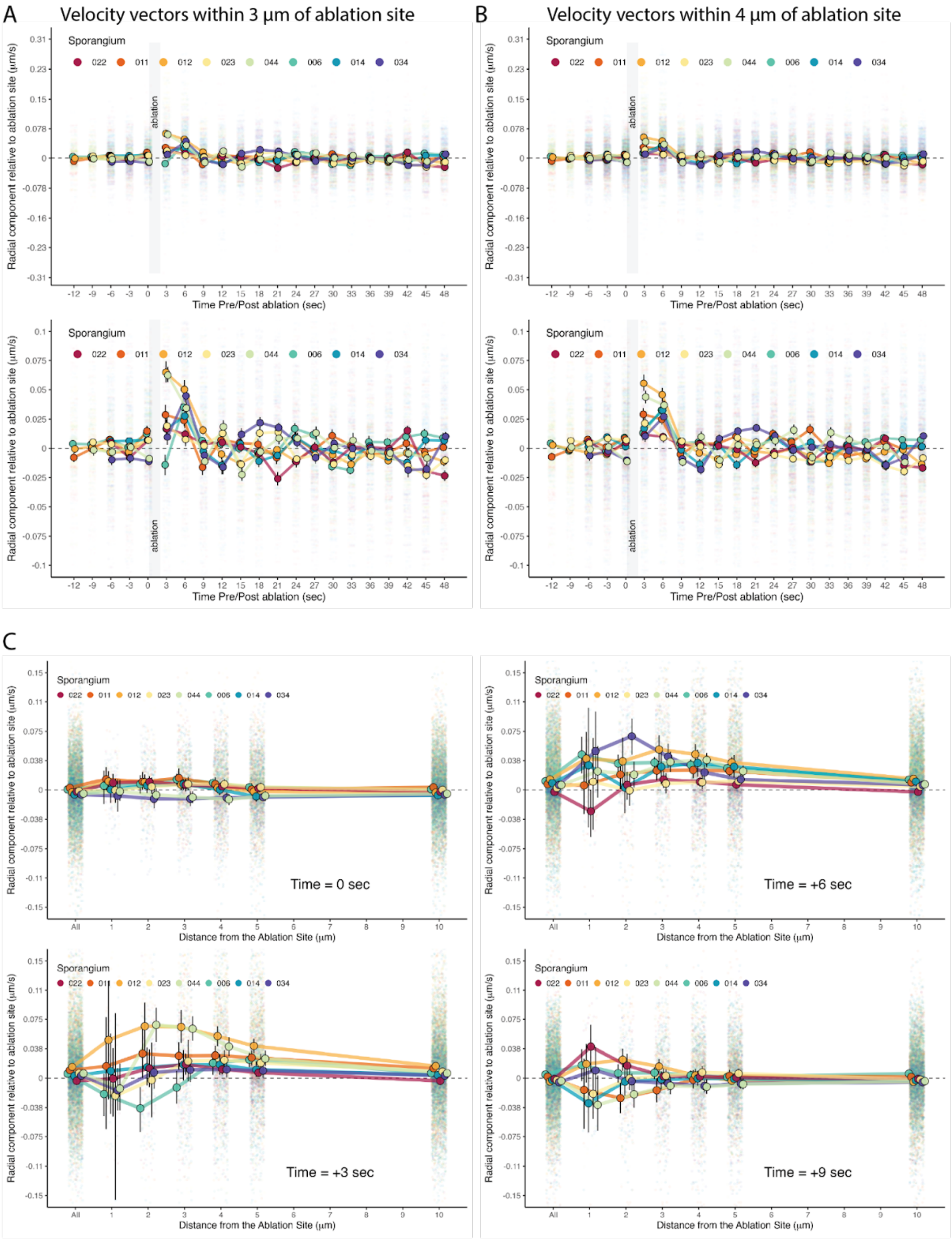

Figure S4. Temporal dynamics, sampling, and distance effects from the ablation site on recoil velocities obtained via PIV. (A & B) PIV retrieved high velocities within 3 µm and 4 µm from the ablation site when using data resampled every 3 seconds. **(A).** Plot showing the radial component relative to the ablation site (velocities away from—positive—or

bioRxiv preprint doi: https://doi.org/10.1101/2025.01.30.635136; this version posted September 24, 2025. The copyright holder for this preprint (which was not certified by peer review) is the author/funder, who has granted bioRxiv a license to display the preprint in perpetuity. It is made available under aCC-BY 4.0 International license.

towards—negative—the ablation site) within 3 μm from the ablation site. Bottom shows an enlarged version of top figure. For visualization purposes we display data points within a range of -0.1 to 0.1 μm/s (this is 111009 points; 95% of the total data). **(B)** Plot showing the radial component relative to the ablation site (velocities away from—positive—or towards—negative—the ablation site) within 4 μm from the ablation site. This is the data shown in Figure 6 in the main text. Bottom panel shows enlarged version of top plot (Figure 6 data). Dots are mean values and error bars show 99% confidence interval of the mean by bootstrap. For visualization purposes we display data points within a range of -0.1 to 0.1 μm/s (this is 176417 points; 95% of the total data). **(C)** Velocities away from or towards the ablation site at increasing distances from the ablation site during 3, 6, and 9 seconds after the beginning of ablation. Top. Plot showing the radial component relative to the ablation site (velocities away from—positive—or towards—negative—the ablation site) just before ablation. “All” uses all velocities within the sporangium that pass signal to noise OpenPIV test and have a magnitude greater than zero. The other plots show distribution of velocities 3,6, and 9 seconds after ablation. Note that the greatest recoil velocities are detected between 2-4 μm microns from the ablation site and 3 seconds after ablation (4 seconds for sporangium 034 & 014). Dots are mean values and error bars show 99% confidence interval of the mean by bootstrap. For visualization purposes 25256 points (99%), 23198 points (97%), 24239 points (98%), and 24628 points (99%) are shown for each plot respectively.
