# Supplementary material for: Three-dimensional cellularization in chytrid fungi uses distinct mechanisms from those driving one- and two-dimensional cytokinesis in animals and yeast
Source: bioRxiv. 2025 Mar 29:2025.01.30.635136. Preprint. [Version 2] doi: 10.1101/2025.01.30.635136 (PMC11974727; doi:10.1101/2025.01.30.635136)
Supplement: Supplementary file 1 [file NIHPP2025.01.30.635136v2-supplement-23.pdf]

bioRxiv preprint doi: https://doi.org/10.1101/2025.01.30.635136; this version posted March 29, 2025. The copyright holder for this preprint (which was not certified by peer review) is the author/funder, who has granted bioRxiv a license to display the preprint in perpetuity. It is made available under aCC-BY 4.0 International license.

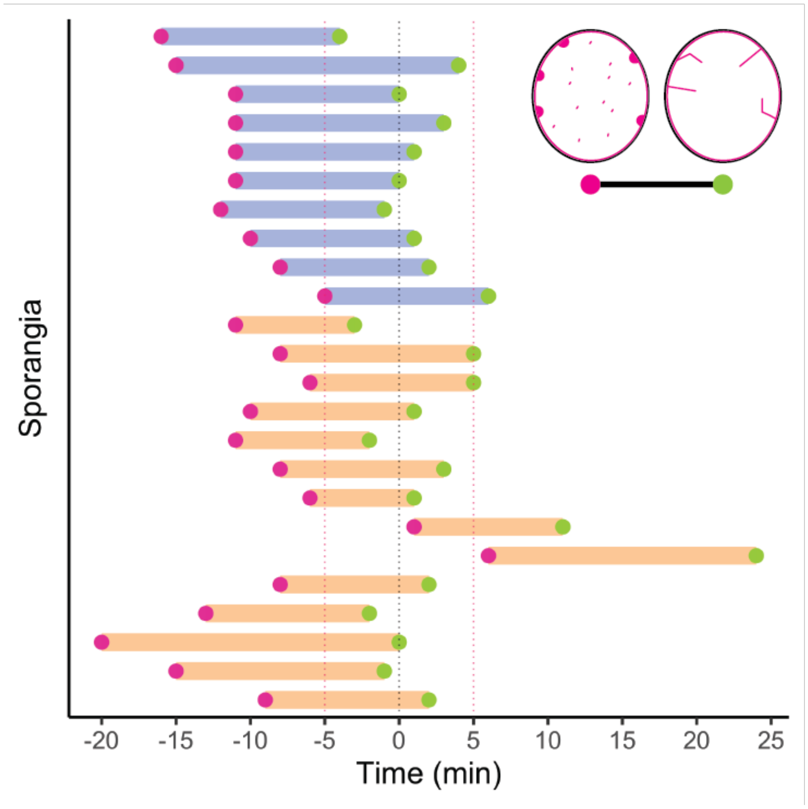


**Figure S1. Furrow ingression coincides with peak cortical nuclear migration** . Each sporangium (row) shows peak cortical nuclear ratio (CNR) at time 0 min. The color bar represent time relative to peak CNR from formation of the first vesicular furrow initial (magenta dot) to the moment the first furrow invades the cytoplasm (green dot). Note how most green dots are within 10 min around peak CNR. Different bar colors represent experiments from different days.

bioRxiv preprint doi: https://doi.org/10.1101/2025.01.30.635136; this version posted March 29, 2025. The copyright holder for this preprint (which was not certified by peer review) is the author/funder, who has granted bioRxiv a license to display the preprint in perpetuity. It is made available under aCC-BY 4.0 International license.

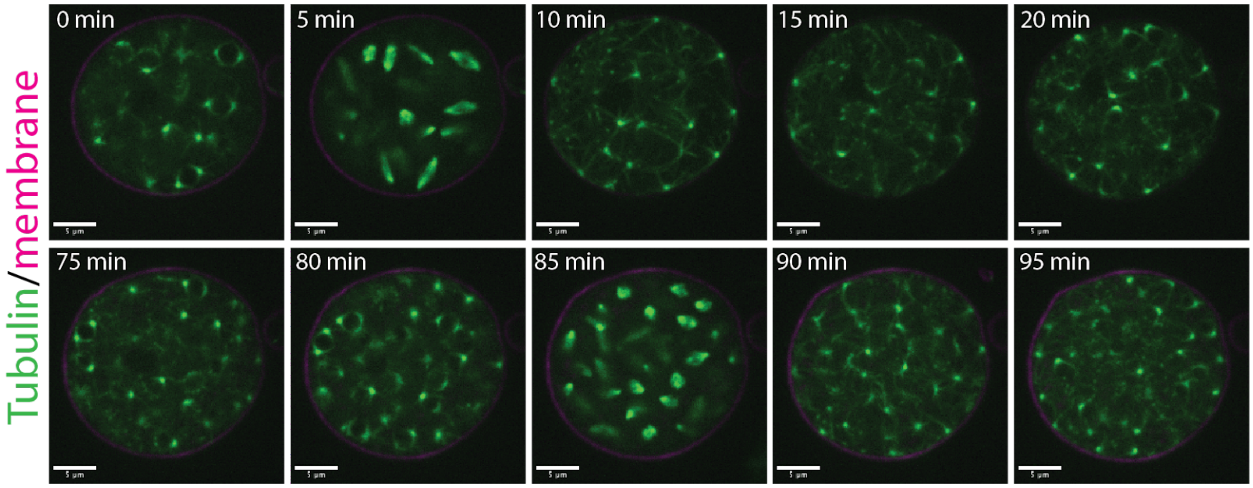


**Figure S2. Nuclear-associated foci of fluorescently-tagged tubulin are likely chytrid centrosomes**. Expression of fluorescently-tagged chytrid alpha-tubulin highlights centrosome-like foci at the poles of each nuclei that turn into opposing spindle poles during nuclear division. Scale bar is 5 microns.

bioRxiv preprint doi: https://doi.org/10.1101/2025.01.30.635136; this version posted March 29, 2025. The copyright holder for this preprint (which was not certified by peer review) is the author/funder, who has granted bioRxiv a license to display the preprint in perpetuity. It is made available under aCC-BY 4.0 International license.

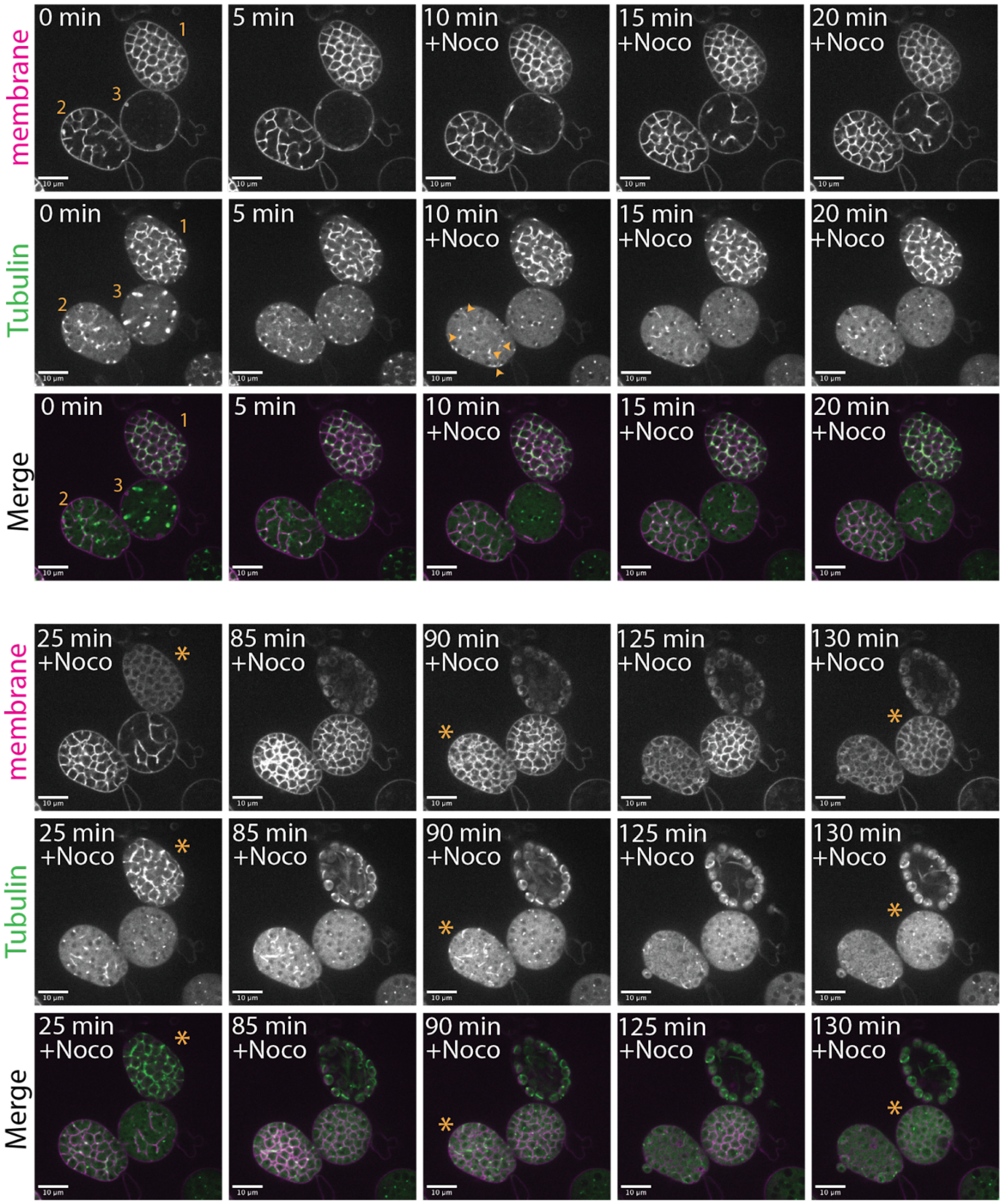


**Figure S3. Cellularization in the presence of Nocodazole does not inhibit cellularization but produces defects in patterning and aciliated daughters**. Example of three mother cells at different stages in cellularization during treatment with Nocodazole (10 min onwards). Note how Nocodazole causes disipation of fluorescent structures except for the bright foci at each nuclei (putative centrosome) and defects in polyhedral patterning. Mother 1 (see yellow numbers) is fully cellularized with daughters fully ciliated. This mother completes cellularization (25 min; asterisk) and daughters escape the mother's cell wall. Mother 2 is at stage 2 of cellularization (branching & merging) and shows some nuclei starting to extend their axoneme (yellow arrows; 10 min) by the time of treatment. This results in a combination of daughters with and without cilia, but still completes cellularization (90 min; asterisk). Mother 3 is at stage 1-2 of cellularization (furrow initiation). Cellularization is completed at 130 min (asterisk) and produces daughter that completely lack cilium. Scale bar 5 microns.

bioRxiv preprint doi: https://doi.org/10.1101/2025.01.30.635136; this version posted March 29, 2025. The copyright holder for this preprint (which was not certified by peer review) is the author/funder, who has granted bioRxiv a license to display the preprint in perpetuity. It is made available under aCC-BY 4.0 International license.

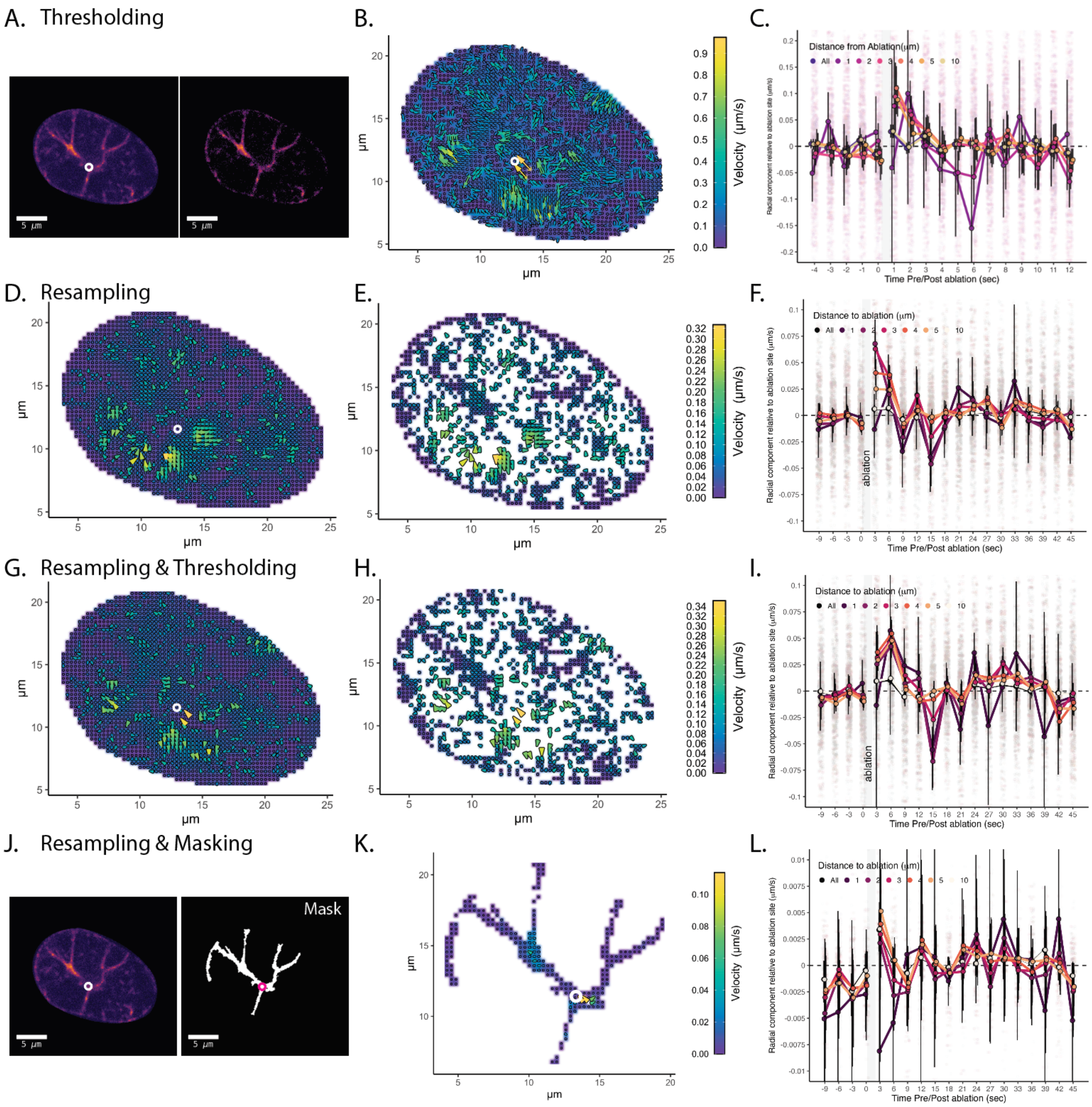


**Figure S4. The signal of recoil after ablation from PIV analysis is robust to different ways of filtering and thresholding the data. (A).** Example images before and after thresholding with Multi-Otsu. **(B)** Flow plot with local velocities estimated by PIV comparing the frame just before ablation (time 0) and the first frame after laser ablation (1 second from the end of ablation)—real time between images is 3 seconds, but time after the end of ablation is about 1 second. **(C)** Plot showing the radial component of velocities relative to the ablation site of all vectors vectors with magnitude above zero and that pass signal to noise test ('peak2peak'; threshold=0.5) from OpenPIV. Dots are mean values and error bars show 99% confidence interval of the mean by bootstrap. For visualization purposes only data within -0.2 and 0.2 μm/s is shown (42573 points; 85.6% of data). **(D)** Flow plot with local velocities estimated by PIV after resampling the image data every 3 seconds such that the complete time series reflect the same sampling rate as before-after laser ablation. **(E)**. Vectors from PIV that pass OpenPIV signal to noise test ('peak2peak'; threshold=0.5) and have a magnitude greater than zero are

bioRxiv preprint doi: https://doi.org/10.1101/2025.01.30.635136; this version posted March 29, 2025. The copyright holder for this preprint (which was not certified by peer review) is the author/funder, who has granted bioRxiv a license to display the preprint in perpetuity. It is made available under aCC-BY 4.0 International license.

the ones used for estimating the radial component of velocities relative to the ablation site. **(F)** Plot showing the radial component of velocities relative to the ablation site for the resampled images. Dots are mean values and error bars show 99% confidence interval of the mean by bootstrap. For visualization purposes only data within -0.2 and 0.2 μm/s is shown (537754 points; 95% of data). **(G)** Flow plot with local velocities estimated by PIV after a combination of resampling and thresholding. **(H)** Vectors from (G) that pass signal to noise and have magnitudes greater than zero. **(I)** Plot showing the radial component of velocities relative to the ablation site for the resampled and thresholded images. Dots are mean values and error bars show 99% confidence interval of the mean by bootstrap. For visualization purposes only data within -0.2 and 0.2 μm/s is shown (49370 points; 92% of data). **(J)** Masking the resampled images to limit the PIV analyses to the furrow regions (based on thresholding of the sum of the images before ablation). **(K)** Flow plot with local velocities estimated by PIV comparing the frame just before ablation (time 0) and the first frame after laser ablation (3 seconds). **(L)** Plot showing the radial component of velocities relative to the ablation site for the resampled and masked images. Dots are mean values and error bars show 99% confidence interval of the mean by bootstrap. For visualization purposes only data within -0.01 and 0.01 μm/s is shown (12042 points; 92% of data).

bioRxiv preprint doi: https://doi.org/10.1101/2025.01.30.635136; this version posted March 29, 2025. The copyright holder for this preprint (which was not certified by peer review) is the author/funder, who has granted bioRxiv a license to display the preprint in perpetuity. It is made available under aCC-BY 4.0 International license.

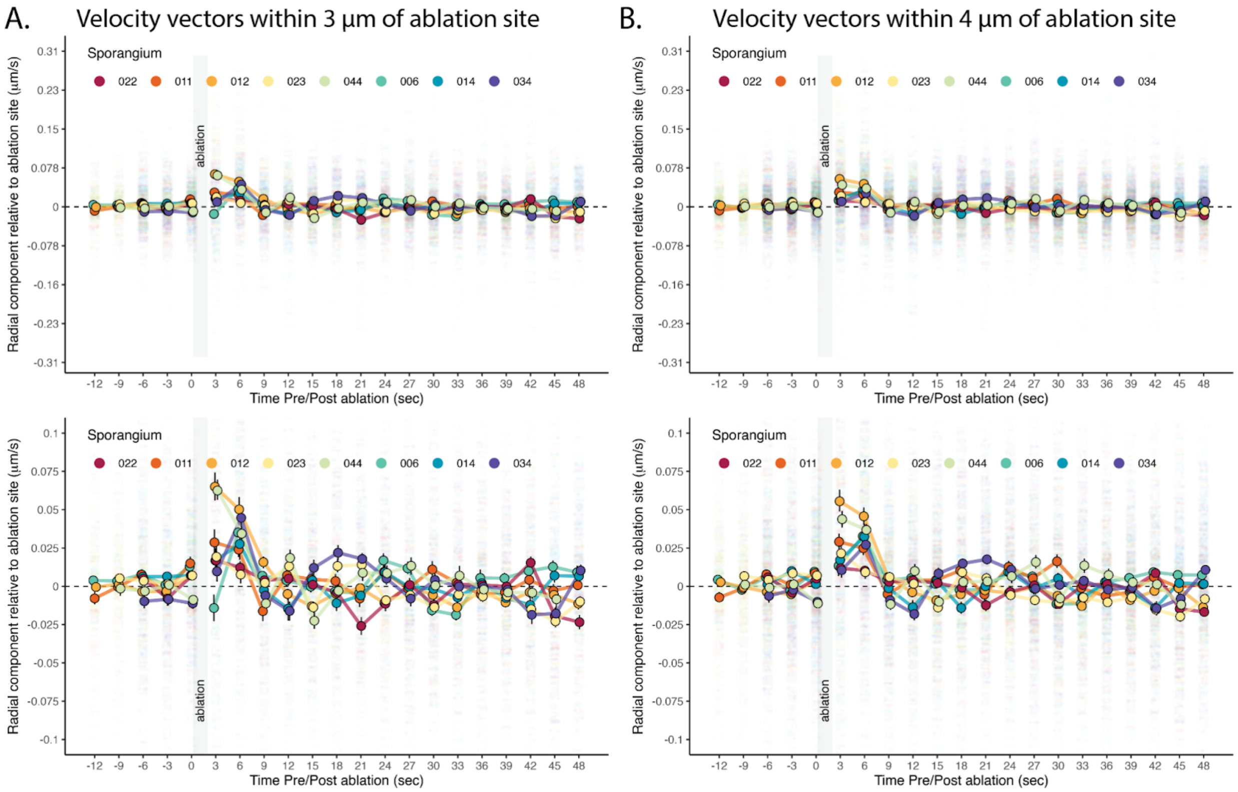


**Figure S5. PIV retrieved high velocities within 3μm and 4μm from the ablation site when using data resampled every 3 seconds. (A).** Plot showing the radial component relative to the ablation site (velocities away from—positive—or towards—negative—the ablation site) within 3μm from the ablation site. Bottom shows an enlarged version of top figure. For visualization purposes we display data points within a range of -0.1 to 0.1 μm/s (this is 111009 points; 95% of the total data). **(B)** Plot showing the radial component relative to the ablation site (velocities away from—positive—or towards—negative—the ablation site) within 4 μm from the ablation site. This is the data shown in Figure 6 in the main text. Bottom panel shows enlarged version of top plot (Figure 6 data). Dots are mean values and error bars show 99% confidence interval of the mean by bootstrap. For visualization purposes we display data points within a range of -0.1 to 0.1 μm/s (this is 176417 points; 95% of the total data).

bioRxiv preprint doi: https://doi.org/10.1101/2025.01.30.635136; this version posted March 29, 2025. The copyright holder for this preprint (which was not certified by peer review) is the author/funder, who has granted bioRxiv a license to display the preprint in perpetuity. It is made available under aCC-BY 4.0 International license.

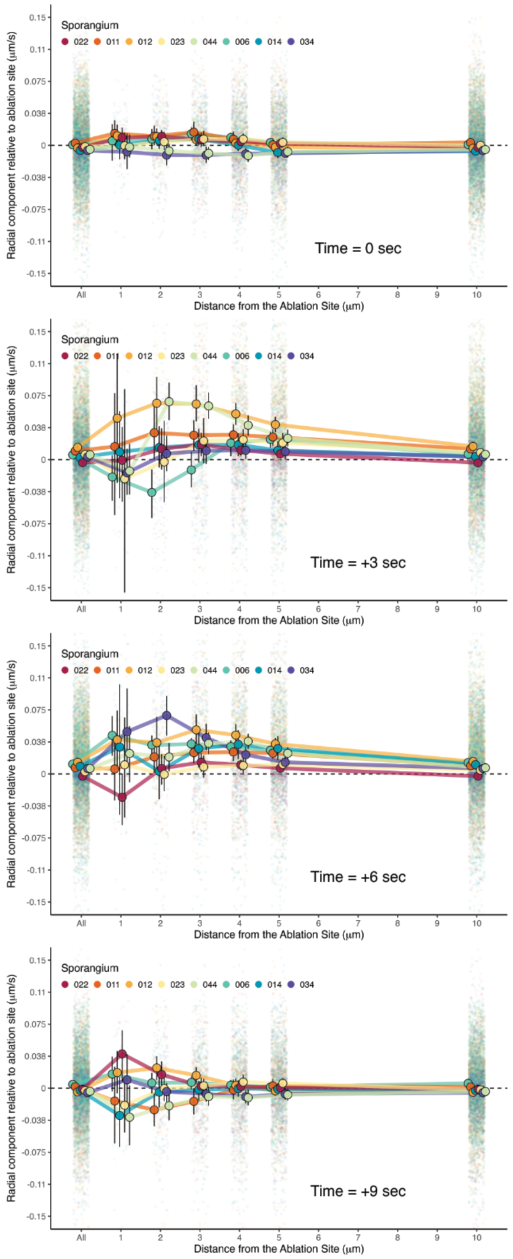


**Figure S6. Velocities away from or towards the ablation site at increasing distances from the ablation site during 3, 6, and 9 seconds after the beginning of ablation.** Top. Plot showing the radial component relative to the ablation site (velocities away from—positive—or towards—negative—the ablation site) just before ablation. “All” uses all velocities within the sporangium that pass signal to noise OpenPIV test and have a magnitude greater than zero. The other plots show distribution of velocities 3,6, and 9 seconds after ablation. Note that the greatest recoil velocities are detected between 2-4 microns from the ablation site and 3 seconds after ablation (4 seconds for sporangium 034 & 014). Dots are mean values and error bars show 99% confidence interval of the mean by bootstrap. For visualization purposes 25256 points (99%), 23198 points (97%), 24239 points (98%), and 24628 points (99%) are shown for each plot respectively.

bioRxiv preprint doi: https://doi.org/10.1101/2025.01.30.635136; this version posted March 29, 2025. The copyright holder for this preprint (which was not certified by peer review) is the author/funder, who has granted bioRxiv a license to display the preprint in perpetuity. It is made available under aCC-BY 4.0 International license.

## SUPPLEMENTARY VIDEO LEGENDS

**Supplementary Video 1. F-actin colocalizes with the cellularization membrane polyehedra of a live chytrid mother cell about to reach peak cellularization.** F-actin (Lifeact-mClover3) shown in green and membrane (FM4-64) in magenta. Note a small section is not completely cellularized. Laser-scanning confocal imaging. Mother cell is about 20 microns deep: Z-plane sweep of 138 Z-stacks, each 0.148 microns. Scale 5 microns.

**Supplementary Video 2. Distribution of nuclei during chytrid peak cellularization.** Nuclei (RbNLS-mClover3) shown in green and membrane (FM4-64) in magenta. Laser-scanning confocal imaging. Mother cell is about 20 microns deep: Z-plane sweep of 130 Z-stacks, each 0.148 microns. Scale 5 microns.

**Supplementary Video 3. Distribution of myosin-II light regulatory chain (MLRC) during chytrid peak cellularization.** A probe of myosin-II activity, MLRC (MLRC-mClover3) in shown in green and membrane (FM4-64) in magenta. Laser-scanning confocal imaging. Mother cell is about 20 microns deep: Z-plane sweep of 136 Z-stacks, each 0.148 microns. Scale 5 microns.

**Supplementary Video 4. Microtubule architecture during chytrid peak cellularization.** Chytrid alpha-tubulin (mClover3-alpha-Tubulin) in shown in green and membrane (FM4-64) in magenta. Laser-scanning confocal imaging. Z-plane sweep of 28 Z-stacks, each 0.6 microns. Scale 5 microns.

**Supplementary Video 5. Nuclear dynamics during chytrid cellularization.** Nuclei visualized with fluorescently-tagged histone H2B (H2B-tdTomato) are shown in green and membrane (FM4-64) in magenta. Spinning-disk confocal time-lapse. Sampling rate of one image per minute. You can see the last two nuclear divisions before the chytrid mother enters cellularization. Scale 5 microns.

**Supplementary Video 6. Example of Nuclear cortical migration during chytrid cellularization.** Nuclei visualized with fluorescently-tagged histone H2B (H2B-tdTomato) are shown in green and membrane (FM4-64) in magenta. Nuclei reach peak cortical ratio around 295 minutes. Spinning-disk confocal time-lapse. Sampling rate of one image per minute. You can see the last two nuclear divisions before the chytrid mother enters cellularization. Scale 5 microns.

**Supplementary Video 7. Example 1 of outward-facing nuclear centrosomes during entry into cellularization.** Fluorescently-tagged tubulin (mClover3-alpha-Tubulin) shown in green and membrane (FM4-64) in magenta. Note how during cortical nuclear recruitment, the nuclear-associated centrosomes are often positioned towards the plasma membrane (100-110 min). Spinning-disk confocal time-lapse. Sampling rate of one image every five minutes. Scale 5 microns.

**Supplementary Video 8. Example 2 of outward-facing nuclear centrosomes during entry into cellularization.** Fluorescently-tagged tubulin (mClover3-alpha-Tubulin) shown in green and membrane (FM4-64) in magenta. Mitotic spindles are visible during nuclear division (85min), and during entry into cellularization (140 min). Note how during cortical nuclear recruitment, the nuclear-associated centrosomes are often positioned towards the plasma membrane (135 min). Nuclei remain tethered to the invading furrow via the centrosome. Spinning-disk confocal time-lapse. Sampling rate of one image every five minutes. Scale 5

bioRxiv preprint doi: https://doi.org/10.1101/2025.01.30.635136; this version posted March 29, 2025. The copyright holder for this preprint (which was not certified by peer review) is the author/funder, who has granted bioRxiv a license to display the preprint in perpetuity. It is made available under aCC-BY 4.0 International license.

microns.

**Supplementary Video 9. Example 3 of outward-facing nuclear centrosomes during entry into cellularization.** Fluorescently-tagged tubulin (mClover3-alpha-Tubulin) shown in green and membrane (FM4-64) in magenta. Note how during cortical nuclear recruitment, the nuclear-associated centrosomes are often positioned towards the plasma membrane (25 min). Nuclei remain tethered to the invading furrow via the centrosome. Spinning-disk confocal time-lapse. Spinning-disk confocal time-lapse. Sampling rate of one image every five minutes. Scale 5 microns.

**Supplementary Video 10. Example 4 of microtubular dynamics during chytrid cellularization.** Fluorescently-tagged tubulin (mClover3-alpha-Tubulin) shown in green and membrane (FM4-64) in magenta. Spinning-disk confocal time-lapse. Spinning-disk confocal time-lapse. Sampling rate of one image every five minutes. Scale 5 microns.

**Supplementary Video 11. Chytrid centrioles appear to dock and tether to the cellularization furrows during chytrid cellularization (Example 1).** Fluorescently-tagged component of the centriole SAS6 (SAS6-mClover3) shown in green and membrane (FM4-64) in magenta. Spinning-disk confocal time-lapse. Spinning-disk confocal time-lapse. Sampling rate of one image every three minutes. Scale 5 microns.

**Supplementary Video 12. Chytrid centrioles appear to dock and tether to the cellularization furrows during chytrid cellularization (Example 2).** Fluorescently-tagged component of the centriole SAS6 (SAS6-mClover3) shown in green and membrane (FM4-64) in magenta. Spinning-disk confocal time-lapse. Spinning-disk confocal time-lapse. Sampling rate of one image every three minutes. Scale 5 microns.

**Supplementary Video 13. Chytrid mothers exposed to Brefeldin-A during late cellularization show collapse of the cellularization furrows.** Fluorescently-tagged tubulin (mClover3-alpha-Tubulin) shown in green and membrane (FM4-64) in magenta. Spinning-disk confocal time-lapse. Sampling rate of one image every three minutes. Scale 5 microns.

**Supplementary Video 14. Chytrid mothers exposed to Brefeldin-A before entry into cellularization fail to enter cellularization.** Fluorescently-tagged tubulin (mClover3-alpha-Tubulin) shown in green and membrane (FM4-64) in magenta. Spinning-disk confocal time-lapse. Sampling rate of one image every three minutes. Scale 5 microns.

**Supplementary Video 15. The actin-based furrows of early cellularization are under tension and recoil where severed by laser ablation (Example 1).** Spinning-disk confocal time-lapse imaging of central plane of a strain expressing the F-actin probe Lifeact-mClover. The actin structures are severed using a laser. Note the fast recoil response after ablation. Scale 5 microns.

**Supplementary Video 16. The actin-based furrows of early cellularization are under tension and recoil where severed by laser ablation (Example 2).** Spinning-disk confocal time-lapse imaging of central plane of a strain expressing the F-actin probe Lifeact-mClover. The actin structures are severed using a laser. Note the fast recoil response after ablation. Scale 5 microns.

**Supplementary Video 17. The actin-based furrows of early cellularization are under tension and recoil where severed by laser ablation (Example 3).** Spinning-disk confocal

bioRxiv preprint doi: https://doi.org/10.1101/2025.01.30.635136; this version posted March 29, 2025. The copyright holder for this preprint (which was not certified by peer review) is the author/funder, who has granted bioRxiv a license to display the preprint in perpetuity. It is made available under aCC-BY 4.0 International license.

time-lapse imaging of central plane of a strain expressing the F-actin probe Lifeact-mClover. The actin structures are severed using a laser. Note the fast recoil response after ablation. Scale 5 microns.

**Supplementary Video 18. The actin-based furrows of early cellularization are under tension and recoil where severed by laser ablation (Example 4).** Spinning-disk confocal time-lapse imaging of central plane of a strain expressing the F-actin probe Lifeact-mClover. The actin structures are severed using a laser. Note the fast recoil response after ablation. Scale 5 microns.

**Supplementary Video 19. The actin-based furrows of early cellularization are under tension and recoil where severed by laser ablation (Example 6).** Spinning-disk confocal time-lapse imaging of central plane of a strain expressing the F-actin probe Lifeact-mClover. The actin structures are severed using a laser. Note the fast recoil response after ablation. Scale 5 microns.

**Supplementary Video 20. The actin-based furrows of early cellularization are under tension and recoil where severed by laser ablation (Example 7).** Spinning-disk confocal time-lapse imaging of central plane of a strain expressing the F-actin probe Lifeact-mClover. The actin structures are severed using a laser. Note the fast recoil response after ablation. Scale 5 microns.

**Supplementary Video 21. The polyhedra of chytrid cellularization are under tension (Example 1).** Spinning-disk confocal time-lapse imaging of central plane of a strain expressing the F-actin probe Lifeact-mClover. The actin structures are severed using a laser. Note the fast recoil response after ablation followed by deformation of the neighboring polyhedra. Scale 5 microns.

**Supplementary Video 22. The polyhedra of chytrid cellularization are under tension (Example 2).** Spinning-disk confocal time-lapse imaging of central plane of a strain expressing the F-actin probe Lifeact-mClover. The actin structures are severed using a laser. Note the fast recoil response after ablation followed by deformation of the neighboring polyhedra. Scale 5 microns.
